# Evolutionary lability in *Hox* cluster structure and gene expression in *Anolis* lizards

**DOI:** 10.1002/evl3.131

**Published:** 2019-08-06

**Authors:** Nathalie Feiner

**Affiliations:** ^1^ Department of Biology Lund University 223 62 Lund Sweden

**Keywords:** Comparative studies, gene expression, Hox genes, Macroevolution, squamates, transposable elements

## Abstract

*Hox* genes orchestrate development by patterning the embryonic axis. Vertebrate *Hox* genes are arranged in four compact clusters, and the spacing between genes is assumed to be crucial for their function. The genomes of squamate reptiles are unusually rich and variable in transposable elements (TEs), and it has been suggested that TE invasion is responsible for the *Hox* cluster expansion seen in snakes and lizards. Using de novo TE prediction on 17 genomes of lizards and snakes, I show that TE content of *Hox* clusters are generally 50% lower than genome‐wide TE levels. However, two distantly related lizards of the species‐rich genus *Anolis* have *Hox* clusters with a TE content that approaches genomic levels. The age distribution of TEs in *Anolis* lizards revealed that peaks of TE activity broadly coincide with speciation events. In accordance with theoretical models of *Hox* cluster regulation, I find that *Anolis* species with many TEs in their *Hox* clusters show aberrant *Hox* gene expression patterns, suggesting a functional link between TE accumulation and embryonic development. These results are consistent with the hypothesis that TEs play a role in developmental processes as well as in evolutionary diversifications.

**Impact Summary**

Although mobile genetic elements are acknowledged to be a driving force of genome evolution, their implications for organismal adaptation and diversification remain poorly understood. One hypothesis is that these transposable elements (TEs) increase the rate of speciation (TEs driving speciation hypothesis). Another hypothesis posits that TEs facilitate the evolution of dramatic changes in morphology, such as seen in snakes (TEs driving innovations hypothesis). This study documents the evolutionary history of TEs in the genomes of snakes and lizards, and tests if invasion of TEs into the developmentally crucial *Hox* gene clusters is associated with changes in how those genes are expressed in embryos. The key findings of this study are:

(1) As other vertebrates, lizards and snakes generally screen off their *Hox* gene clusters from TE invasions.
(2) The extraordinarily species‐rich *Anolis* lizards are an exception, and TE content of *Hox* clusters of some *Anolis* species approach genome‐wide levels.
(3) The age distribution of TEs in *Anolis* lizards suggests that peak TE activity coincides with speciation events (consistent with the TEs driving speciation hypothesis).
(4) Lizards with large numbers of TEs in their *Hox* clusters show aberrant *Hox* gene expression patterns. This suggests that TEs may alter regulation of developmental genes (consistent with the TEs driving innovations hypothesis).

Together, these results support a role for mobile genetic elements as potential engines of evolvability. TEs accumulate with speciation events and affect the regulation of development, thereby shaping the raw material for evolutionary change.

Across the animal kingdom, *Hox* genes specify positional identities along the head‐to‐tail body axis (Kmita and Duboule 2003; Deschamps and van Nes 2005). Nested expression patterns of *Hox* genes are interpreted by the downstream developmental system, and thus form the basis for morphological patterning in early development. In vertebrates, *Hox* genes are arranged in four compact gene clusters (HoxA–D), derived from two duplications of a single prevertebrate cluster (Holland et al. 1994; Lemons and McGinnis 2006). This organization is responsible for the spatiotemporal expression pattern of *Hox* genes, such that the position of a given *Hox* gene in the cluster defines its time and domain of expression in the developing embryo (Duboule and Dolle 1989; Graham et al. 1989; Duboule 2007). This is known as the *Hox* code (Gaunt 1994). In tetrapods, a subset of *HoxA* and *HoxD* genes gained an additional task in patterning of the limb, co‐opting the collinear mode of expression along the head‐to‐tail axis (Tarchini and Duboule 2006).

Most knowledge about the regulation and function of *Hox* genes is derived from studies of model organisms, in particular mice and to a lesser extent chickens. The degree to which *Hox* cluster structure and *Hox* gene expression are conserved across tetrapods is therefore largely unknown. Recent comparative data suggest that the genomes of squamate reptiles (lizards and snakes) are fundamentally different to those of birds and mammals by being particularly rich and variable in the content of transposable elements (TEs; Pasquesi et al. 2018). In line with this finding, at least some squamates have enlarged *Hox* clusters (Di‐Poï et al. 2009, 2010; Woltering et al. 2009; Woltering 2012; Feiner 2016). This is hypothesized to be caused by the accumulation of TEs, which are generally excluded from *Hox* clusters in birds and mammals (Di‐Poï et al. 2009, 2010). Comparative evidence from the species‐rich lizard genus *Anolis* suggests that TEs in *Hox* clusters accumulate with speciation events, resulting in a highly variable length of *Hox* clusters even within this genus (Feiner 2016). Although it is tempting to speculate that genome‐wide characteristics of the repeat landscape dictate *Hox* cluster structure (length and TE content), this has not been formally tested.

If spatial and temporal collinearity of *Hox* gene expression is indeed maintained by close physical proximity of *Hox* genes in a compact cluster (Ferraiuolo et al. 2010; Mallo and Alonso 2013; Papageorgiou 2017), invasion of TEs could interfere with *Hox* cluster function by increasing intergenic distances between *Hox* genes. Insertion of TEs could also introduce novel cis‐regulatory elements (Slotkin and Martienssen 2007; Feschotte 2008; Cowley and Oakey 2013; Sundaram et al. 2014), or affect chromatin modifications (Kidwell and Lisch 1997). The limited data on *Hox* gene expression patterns in lizards suggest that these largely follow the typical tetrapod *Hox* code; *HoxA11* and *HoxA13* of *Anolis sagrei* and *Anolis angusticeps* (Wakasa et al. 2015), and *HoxA10*, *‐B10*, *‐C10*, and *‐13* genes of the whiptail lizard *Aspidoscelis uniparens* (Di‐Poï et al. 2010) conform to the normal expression pattern for tetrapods, whereas *HoxD10* expression of the whiptail lizard has shifted its boundary and does not mark the lumbo‐sacral transition, as it is typical for other tetrapods (Di‐Poï et al. 2010).

The highly derived body plan of snakes has motivated research to establish if their morphology is accompanied, or indeed caused, by aberrant *Hox* gene expression patterns (Cohn and Tickle 1999; Woltering et al. 2009; Di‐Poï et al. 2010; Head and Polly 2015). In the python, *HoxC6* and *‐C8* show an expansion of expression patterns along the primary body axis, whereas *HoxB5* is expressed similarly to other tetrapods (Cohn and Tickle 1999). In the corn snake *Pantherophis guttatus*, 13 *Hox* genes show the expected tetrapod expression patterns (Woltering et al. 2009; Di‐Poï et al. 2010), whereas the anterior boundaries of *HoxA10* and *‐C10* genes have shifted relative to the tetrapod norm, and *HoxA13* and *‐D13* have lost expression in the post‐cloacal tail bud (Di‐Poï et al. 2010). These shifts and losses of *Hox* expression domains have been hypothesized to be the result of an alternative interpretation of the *Hox* code for the axial patterning of snakes (Woltering et al. 2009; but see Head and Polly 2015). However, the available data on *Hox* gene expression patterns in squamates are too sparse to enable reliable inferences on the extent to which TE‐derived expansions of *Hox* clusters are accompanied by changes in gene expression patterns.

To address these gaps in our knowledge, I studied the structural organization of *Hox* clusters in 10 snake and 12 lizard species, while explicitly considering genome‐wide characteristics. Although snakes are nested within the paraphyletic group of lizards, previous research motivates a specific comparison between the two groups. I apply a methodology specifically developed to alleviate biases inherent in the annotation of TEs with similarity‐based identifications, and thus offer a comprehensive survey of the TE landscapes of squamate *Hox* clusters. I also assessed the potential impact of high TE densities within *Hox* clusters by comparing *Hox* gene expression patterns during embryonic development in a species with moderate *Hox* cluster TE density (a Lacertid lizard, the common wall lizard, *Podarcis muralis*) and in three species of *Anolis* lizards, where *Hox* cluster TE density is highly variable and TE content is particularly high for lineages with high rates of speciation (Feiner 2016). I therefore compared one *Anolis* species with low *Hox* cluster TE density (the West Cuban anole, *Anolis bartschi*; Feiner 2016) with two *Anolis* species from distantly related clades with high *Hox* cluster TE densities (the brown anole, *A. sagrei* and the green anole, *A. carolinensis*).

Overall, this study shows that *Anolis* lizards are characterized by the longest and most TE‐rich *Hox* clusters, and that lizards generally have more TE‐rich and longer *Hox* clusters than snakes. The amount of TEs in a *Hox* cluster, and not genome size, is the best predictor of *Hox* cluster size, and *Hox* cluster TE content is approximately 50% lower than genome‐wide TE content. However, *Hox* cluster TE content approaches genome‐wide TE content in two *Anolis* species. These two species with TE‐rich *Hox* clusters, but no species with TE‐poor *Hox* clusters, show aberrant expression patterns for one out of the four tested posterior *Hox* genes. This indicates that further mechanistic and comparative studies of squamates will be able to reveal if expansions of *Hox* clusters are responsible for changes in the expression patterns of *Hox* genes.

## Materials and Methods

### RETRIEVAL OF GENOMIC SEQUENCES AND IDENTIFICATION OF *HOX* CLUSTERS

I screened the literature and public databases for genome‐wide sequence resources of squamate reptiles. All available data were downloaded as fasta files without annotations or maskings and information of genome sizes from accompanying descriptions was collected (Table S1). To identify *Hox* clusters, flanking genes of each *A. carolinensis Hox* cluster (*HoxA1*, *‐A13*, *‐B1*, *‐B13*, *‐C1*, *‐C13*, *‐D1*, and *‐D13* as annotated in Ensembl Release 91; Hubbard et al. 2009) were used as queries in tblastn searches against all genomes. I used reciprocal blastx searches against nonredundant protein sequences in NCBI to verify the identity of individual *Hox* genes. If the two blast hits for a given paralog group in a species were located on the same scaffold, that is, if the *Hox* cluster sequence was contiguous in the genome assembly, the *Hox* cluster sequence was extracted using BEDTools software version 2.27.1 (Quinlan and Hall 2010).

### ANNOTATION OF TRANSPOSABLE ELEMENTS

Genomic TEs can be detected either by applying similarity‐based searches against a reference TE library, or by de novo prediction of repetitive sequences within a genome. The former strategy is sensitive to the taxonomic composition of the reference TE library, in particular, if the aim is to compare TE contents between species whose representation in the reference TE library is skewed. For example, the vertebrate Repbase library version RepBase23.01 (Jurka 2000) consists of a total of 13,409 entries, and out of 406 entries for lepidosauria, 371 entries are derived from the *A. carolinensis* genome. Using this library as reference in similarity‐based TE annotation (using RepeatMasker software version 4.0.7; Smit et al. 2013) results in a TE detection bias toward *A. carolinensis* and congeners (Fig. S1). Therefore, I opted for a strategy applying de novo prediction of TEs per species, which alleviates a species bias and in addition allows the detection of novel TEs. In brief, I applied the RepeatModeler software version 1.0.11 (Smit and Hubley 2008), which implements the repeat finding tools RECON (Bao and Eddy 2002) and RepeatScout (Price et al. 2005). The predicted species‐specific TE libraries were used as references in RepeatMasker searches using *Hox* cluster sequences as well as whole‐genome sequences of the focal species as target. I extracted the percentages of the *Hox* clusters and genomes that are covered by TEs from RepeatMasker output files. These output files were also used to retrieve chromosome/scaffold distributions of TEs in window sizes of 500 kb (Figs. S2 and S3). For TE class‐specific analyses, the classification system implemented in RepeatMasker was used.

I visualized the repeat landscapes of *Hox* clusters as well as whole genomes by plotting the distribution of Kimura substitution levels (a proxy for TE age) using the accompanying perl scripts in the RepeatMasker utility.

### ANIMAL HUSBANDRY

To assess the level of conservation in expression profiles of posterior *Hox* genes (*Hox13*), I visualized their gene expression patterns in four species of lizards; a Lacertid (the common wall lizard, *P. muralis*) and three congener Iguanids (the West Cuban anole *A. bartschi*, the brown anole *A. sagrei*, and the green anole *A. carolinensis*). I also stained expression patterns of the focal genes in mouse embryos. I focused the analyses on “limb‐bud” embryonic stages to reduce the temporal dimension in the comparative framework.

*Anolis bartschi*, *A. sagrei*, and *A. carolinensis* lizards were bred in‐house in groups of one male and one to four females per cage (590 × 390 × 415 mm). Cages were equipped with a plant, twigs, and bast mats as shelter. Lizards were kept at a light cycle of 12 L:12 D, and given access to basking lights (60 W) for 8 hours per day and a UV light (EXO‐TERRA 10.0 UVB fluorescent tube) for 4 hours per day. Mealworms and crickets were provided ad libitum. Pots filled with soil for egg laying were provided and checked daily during the breeding season. Wall lizards were wild caught from Italy and France and kept under the same light conditions, but cages were equipped with sand as substrate and bricks as shelter. Cages of female wall lizards were inspected daily for nesting sites. All eggs were incubated at 26°C in 0.2 l plastic cups filled two‐thirds with moist vermiculite (5:1 vermiculite/water volume ratio) and sealed with clingfilm.

Eggs were dissected in nuclease‐free phosphate‐buffered saline (PBS) and immediately fixed in 4% paraformaldehyde (PFA) at 4°C for 12–24 hours. Embryos were transferred to methanol through a dilution series in PBS and kept at –20°C for storage. A subset of embryos was transferred to RNAlater (Qiagen) immediately following dissection to stabilize RNA for the isolation of *Hox* genes (see below). Mouse embryos were obtained from collaborators, fixed in 4% PFA and stored in methanol, or submerged in RNAlater. I report the stage of all lizard embryos according to Dufaure and Hubert (1961) because the staging table for *Anolis* lizards (Sanger et al. 2008) provides lower resolution.

### GENE EXPRESSION ANALYSIS

To analyze gene expression patterns using *in situ* hybridizations, I prepared RNA probes for *Hox* genes of the Hox13 paralog groups for the four lizard species and mouse. Total RNA from a single embryo of a common wall lizard (*P. muralis*, stage 27), a West Cuban (*A. bartschi*), a brown (*A. sagrei*), and a green anole (*A. carolinensis*, all stage 28) and a mouse (9.5 days postfertilization) were extracted and reverse transcribed into cDNA using SuperScript III (Invitrogen, Carlsbad, US), following the instructions of the 3′‐RACE System (Invitrogen, Carlsbad, US). Oligonucleotide primers were designed based on genomic sequences to amplify partial cDNAs of *Hox* genes. Because the West Cuban anole lacks genomic resources, cDNAs were first amplified using primers designed for congeners with known sequence data. In a second step, species‐specific primers were designed to obtain probes against West Cuban anole *Hox13* genes. Details on isolated fragments and primer sequences are provided in Table S2.

The obtained *Hox* fragments were used as templates for riboprobe synthesis using the DIG RNA labeling kit (Roche, Mannheim, Germany). Whole‐mount *in situ* hybridizations were performed using embryonic wall lizards, West Cuban, green and brown anoles, and mice with RNA probes against *Hox13* paralog group genes. I followed the protocol of Woltering et al. (2009) with the modification that the triethanolamine treatment step was omitted. Expression patterns were documented with a Nikon microscope (Nikon SMZ18) using the imaging software NIS‐Elements BR 5.02.00. Observed expression patterns for each gene were confirmed in independent experiments on at least three mouse embryos, at least three *P. muralis* embryos, and at least five embryos of each *A. carolinensis* and *A. sagrei*. Scarcity of *A. bartschi* embryos restricted the gene expression analysis to each one embryo for *HoxA13*, *‐B13*, and *‐C13*, and three embryos for *HoxD13*.

## Results

### *HOX* CLUSTER SIZE

By screening 22 squamate genomes (10 snakes and 12 lizards), 17 genome assemblies were identified that contain at least one contiguous and complete *Hox* cluster, totaling 49 squamate *Hox* clusters (Table S1). Insufficient quality of sequence assemblies explains the absence of contiguous clusters, as those were more frequent in low‐quality genomes (Table S1). *Hox* clusters of *Anolis* species (e.g., *A. bartschi*) identified in a previous study were not included because they cover only partial clusters and lack genome‐wide sequence information (Feiner 2016).

**Figure 1:**
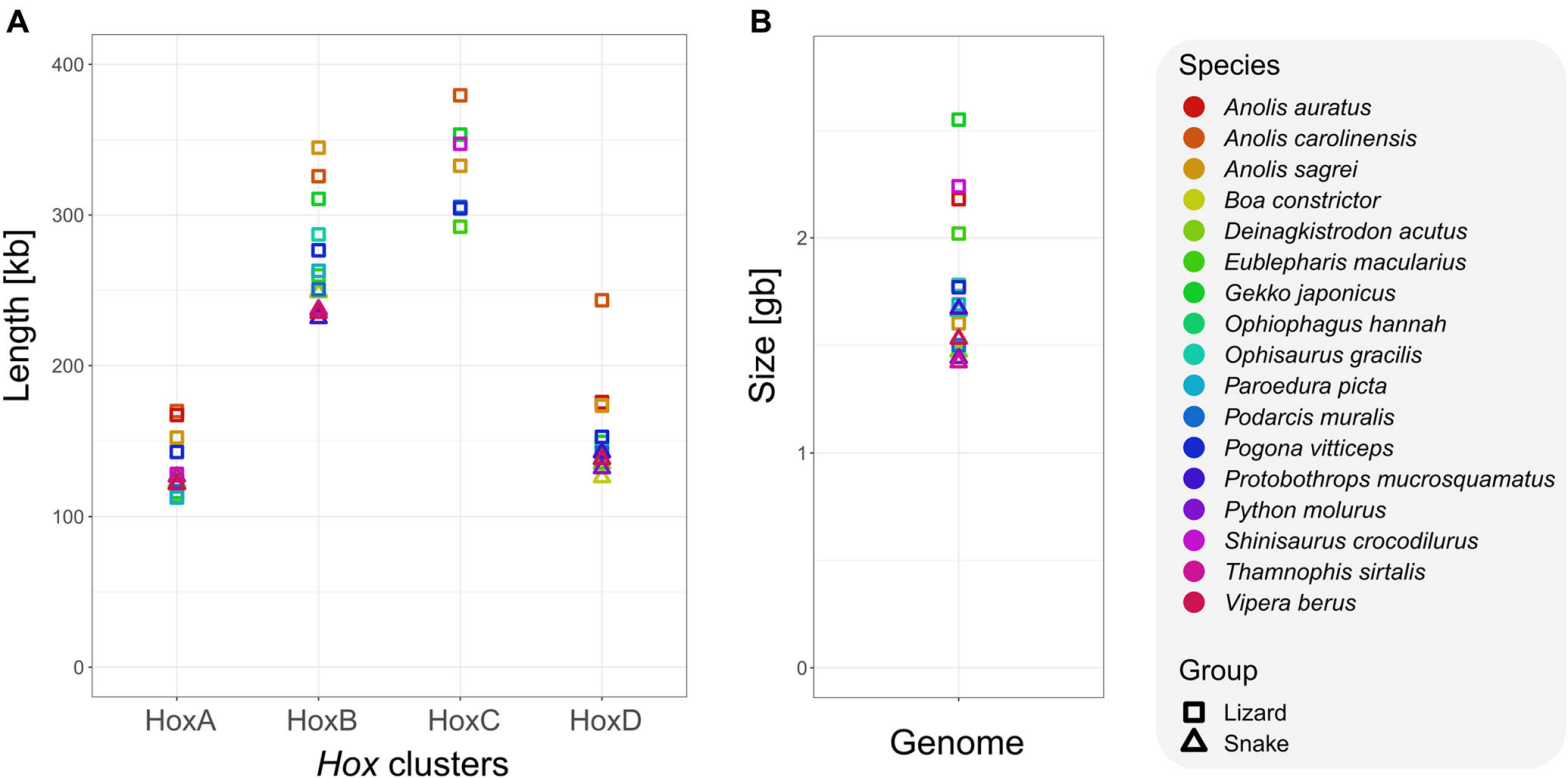
*Hox* cluster length and genome sizes in squamate reptiles. (A) Raw lengths of *Hox* clusters and (B) genome sizes are plotted for 17 squamate species. For all four *Hox* clusters, except for the HoxC cluster, the cluster lengths of *Anolis* lizards are larger than those of other squamates despite that their genome sizes are moderate. Lizards have generally larger *Hox* clusters and larger genomes than snakes.

Individual *Hox* clusters of *Anolis* lizards (the four clusters of *A. carolinensis* and *A. sagrei*, and the HoxA and ‐D clusters of *A. auratus*) are longer than *Hox* clusters in other squamates (Fig. 1A). An alignment of *A. carolinensis Hox* clusters to moderately sized *Hox* clusters of *P. muralis* reveals that sizes of *Hox* gene bodies (coding sequences plus introns) are largely conserved between the two species, and that the elongation of *A. carolinensis Hox* clusters is evenly distributed across intergenic regions (Fig. S4). *Hox* cluster length is not explained by genome size (phylogenetic generalized least squares [PGLS] regression of genome size on cluster length: HoxA, *P* = 0.59, HoxB, *P* = 0.43, HoxC, *P* = 0.19, HoxD, *P* = 0.85). Although *Anolis* lizards have the largest *Hox* clusters, their genome sizes are moderate (Fig. 1B).

### CONTENT OF TRANSPOSABLE ELEMENTS INSIDE AND OUTSIDE OF *HOX* CLUSTERS

**Figure 2:**
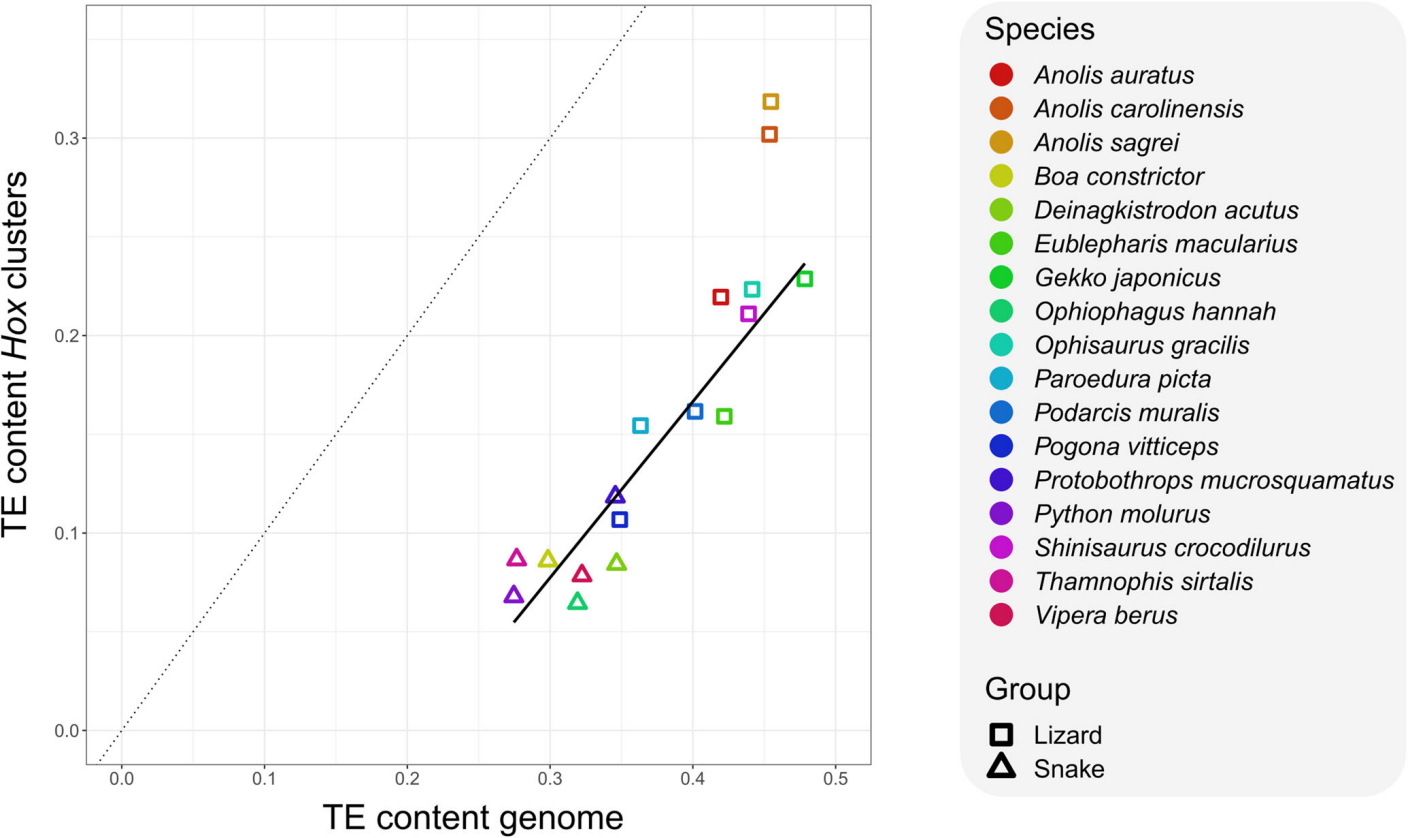
Relationship between TE content in *Hox* clusters and whole genomes in squamate reptiles. De novo predicted TE contents for each species are plotted. All squamates, except for *Anolis carolinensis* and *A. sagrei*, follow a linear trend with TE content in *Hox* clusters increasing with the amount of TE across the genome. Given the genome‐wide TE content, *Hox* clusters remain relatively TE poor. If TEs would not be restricted from *Hox* cluster, we would expect a 1:1 relationship between TE content in *Hox* clusters and genome‐wide TE content (dashed line). Regression line (solid line) was fitted for squamates excluding *A. sagrei* and *A. carolinensis* and is only included for graphical purpose.

The amount and type of TEs residing in a genome are a decisive factor for genomic characteristics. In the dataset of 17 squamate genomes, genome‐wide TE content is weakly correlated with genome size (PGLS regression of TE content on genome size, *P* = 0.09). If there was no mechanism for restricting TEs from *Hox* clusters, one would expect a 1:1 relationship between the TE content of *Hox* clusters and genome‐wide TE content (dashed line in Fig. 2). The TE content of squamate *Hox* clusters increases linearly with genome‐wide TE content, generally reaching about 50% of the genome‐wide TE content. In contrast, *A. carolinensis* and *A. sagrei*, but not *A. auratus*, have unusually TE‐rich *Hox* clusters, approaching 75% of the genome‐wide TE content (Fig. 2). Lizards have consistently higher TE contents than snakes in both their genomes (42% in lizards vs. 31% in snakes) as well as their *Hox* clusters (21% in lizards vs. 8% in snakes; Fig. 2).

### TE CLASSES AND DYNAMICS

**Figure 3:**
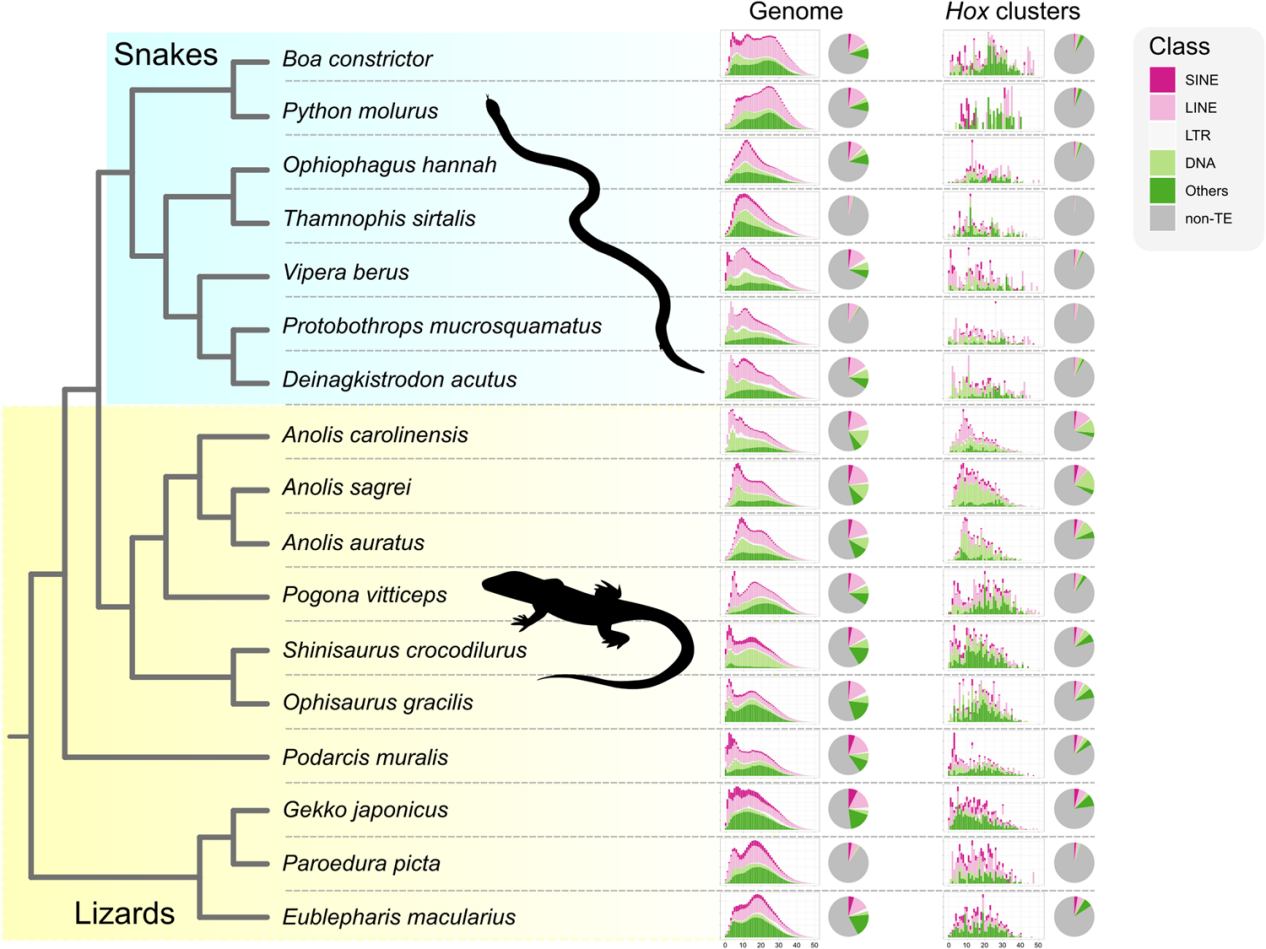
Comparison of TE landscapes between *Hox* clusters and genomes of squamate reptiles. Left side of the figure shows schematically the phylogenetic relationships among species included in this study. Right side of the figure shows metrics of TE age distributions (bar plots) and TE contents (pie charts) per species, both for whole‐genomes and *Hox* clusters. Bar plots show the frequencies of Kimura substitution levels (a proxy for TE age) for de novo predicted TEs at an arbitrary scale. Pie charts show the overall TE‐content and the composition of different repeat classes. Pie charts illustrate lower overall TE content in snake genomes as well as *Hox* clusters, similar to the trend shown in Figure 2. TE landscapes (bar plots) show species‐specific trends in TE dynamics, for example, a recent activity of SINEs in the common European adder *Vipera berus* and *P. muralis*.

Given the enrichment of TEs in the *Hox* clusters of *A. carolinensis* and *A. sagrei* compared to other squamates, I next investigated whether this pattern is driven by the invasion of a particular class of TEs. I inspected the composition of TEs across genomes and in *Hox* clusters at the level of TE classes, and created TE landscapes to assess the age‐distribution across TE classes. This revealed marked differences across species, with generally more heterogeneous TE landscapes in snakes (Fig. 3). Genome‐wide TE landscapes of *A. carolinensis* and *A. sagrei* are largely dominated by long interspersed nuclear elements (LINEs) and DNA transposons. Compared to other squamates, DNA transposons appear to be slightly overrepresented in the *Hox* clusters of both *A. carolinensis* and *A. sagrei* (Fig. 3).

Because TE content across the genome is rather heterogeneous (particularly centromeric and telomeric regions), TE content in the immediate neighborhood of *Hox* clusters was visualized. This revealed that the regions containing *Hox* clusters in the genomes of *A. carolinensis* and *A. sagrei* are equally, or more, TE‐rich than their chromosomal neighborhood (Fig. S2). In contrast, *Hox* clusters of other lizards reside in genomic locations that are TE impoverished compared to neighboring chromosomal regions (Fig. S2). When considering different TE classes across the entire *Hox* cluster‐containing chromosomes of *A. carolinensis* and *A. sagrei*, LINE elements are enriched in centromeric and telomeric regions, whereas short interspersed nuclear elements (SINEs) show the opposite pattern (Fig. S3).

### TES AND PATTERNS OF DIVERSIFICATION

The TE content of *Hox* clusters has been shown to positively correlate with the number of speciation events in lineages of *Anolis* lizards (Feiner 2016). The broad, but sparse, taxonomic coverage and poorly resolved phylogenies for some taxa prevents a global test of a relationship between speciation events and TE invasion. Following the results of Feiner (2016), the analysis was therefore restricted to *Anolis* lizards and asked if TEs in *Hox* clusters accumulated continuously over time, or if the invasion was a temporally restricted event.

The three *Anolis* lizards in this dataset belong to lineages with markedly different histories of diversification frequencies. Although the lineages leading to *A. carolinensis* and *A. sagrei* experienced, respectively, seven and five speciation events in the past 30 million years, *A. auratus* experienced only a single one in the same period (Poe et al. 2017). TE landscapes of *A. carolinensis*, and to a lesser extent *A. sagrei*, show a shift toward higher frequencies of young TEs (low Kimura substitution levels) relative to *A. auratus* (Fig. 3). This trend is evident both in the genome as well as in the *Hox* cluster, and persists when TE landscapes are time calibrated with an estimated substitution rate of the genus *Anolis* (Tollis et al. 2018; Fig. S5). I find that *A. auratus*—an anole with TE‐impoverished *Hox* clusters relative to other congeners—is characterized by few recent speciation events and little recent TE activity, whereas *A. carolinensis* underwent many speciation events in the past 30 million years and possesses large numbers of young, active TEs (Figs. 3 and S5). Thus, the frequency distribution of TEs over age classes is broadly coinciding with the estimated timing of speciation events in the three *Anolis* lineages.

### EXPRESSION OF *HOX* GENES IN RELATION TO TE CONTENT

**Figure 4:**
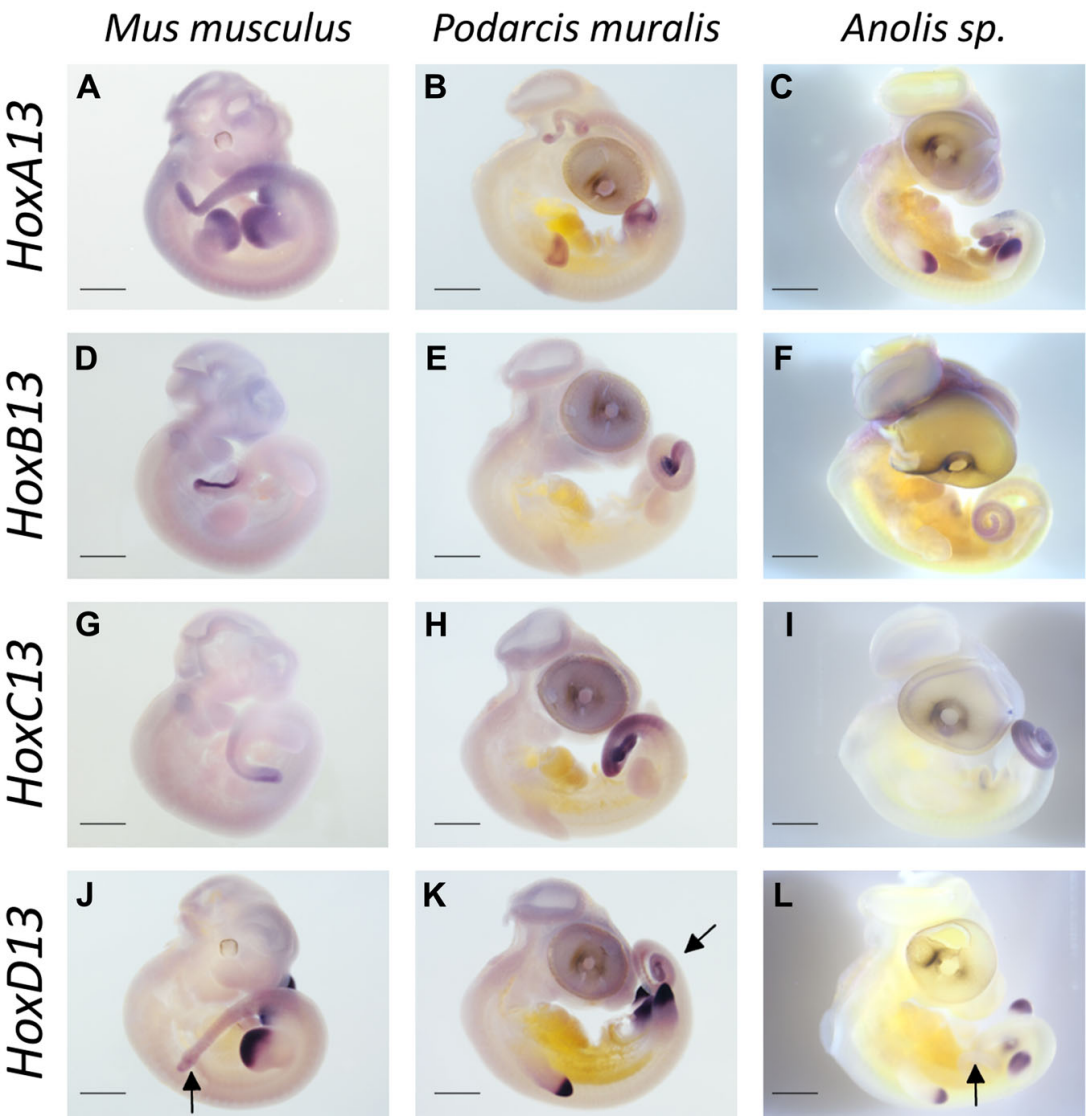
Expression patterns of Hox13 paralog group genes in embryonic *Mus musculus* (stage E 10.5), *Podarcis muralis*, and *Anolis sp*. (all stage 32). (A–C) *HoxA13* is expressed in fore‐ and hindlimb buds, tail tissue, and genitalia (not visible). (D–F) *HoxB13* is expressed in the tail tip, but absent from limb buds. (G–I) *HoxC13* is expressed in tail tissue. (J–L) *HoxD13* is expressed in fore‐ and hindlimb buds of all species investigated, and in tail tissue of *Mus musculus* and *Podarcis muralis*, but variably expressed in *Anolis* lizards (absent in *A. sagrei* shown in panel l). See Figure 4 in main text for more information on *HoxD13* expression in *Anolis* species. Arrows indicate differences in expression domains. Expression patterns of *HoxA13*, *HoxB13*, and *HoxC13* are conserved between all *Anolis* species examined in this study (*A. sagrei* is shown). Scale bars, 100 µm.

**Figure 5:**
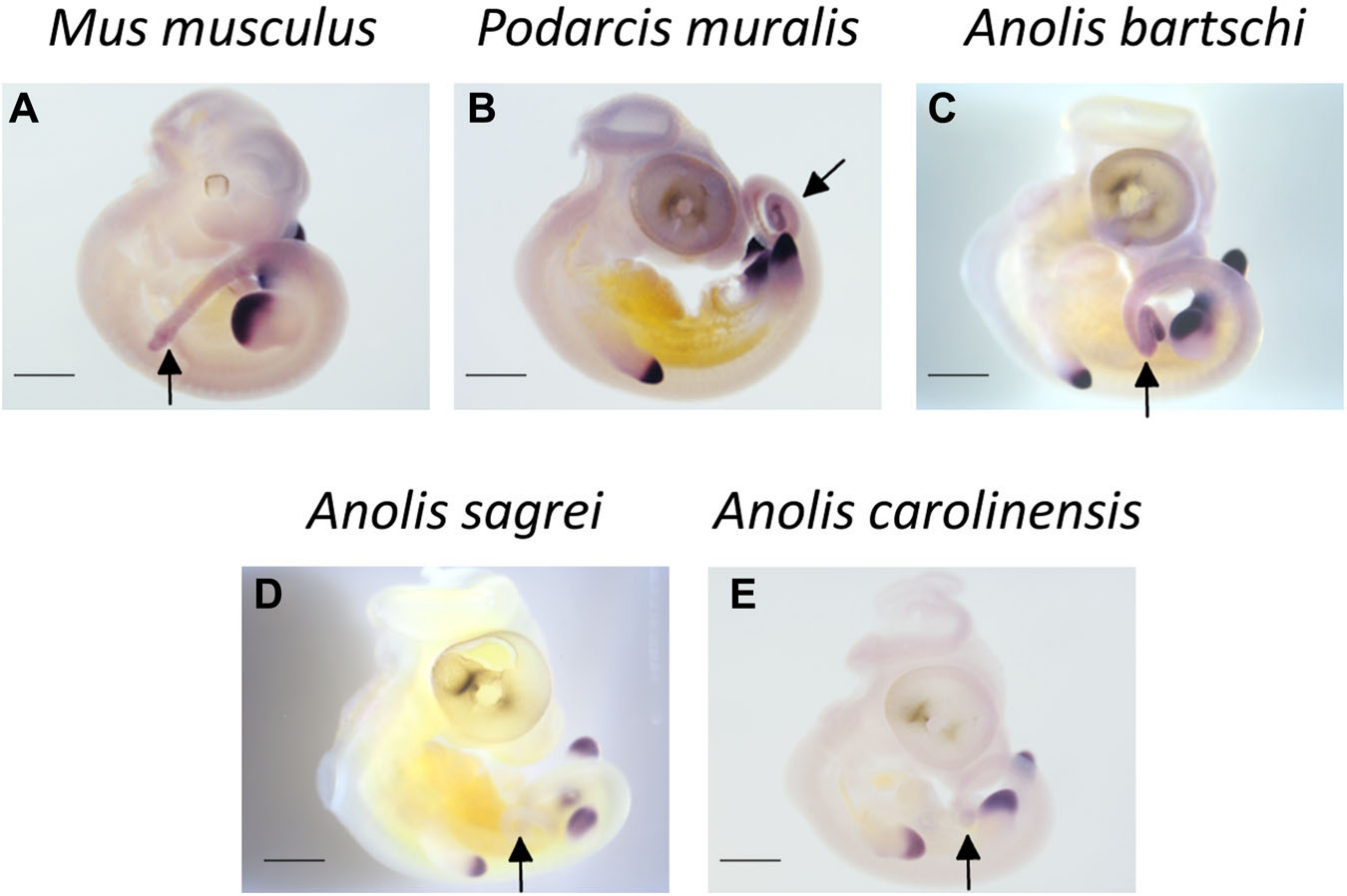
Expression patterns of *HoxD13* genes in embryonic *Mus musculus* (stage E 10.5), *Podarcis muralis*, *Anolis bartschi*, *A. sagrei*, and *A. carolinensis* (all stage 32). (A–C) *HoxD13* is expressed in fore‐ and hindlimb buds and tail tissue in *M. musculus*, *P. muralis*, and *A. bartschi*. (D and E) *HoxD13* is expressed in fore‐ and hindlimb buds in *A. sagrei* and *A. carolinensis*. Arrows show presence (A–C) and absence (D and E) of *HoxD13* expression in tail tissue. Scale bars, 100 µm.

To investigate the level of conservation of expression patterns between *Hox* genes derived from *Hox* clusters with radically different TE contents, we compared expression domains of all *Hox13* paralog group genes in a corresponding developmental stage between selected species. The mouse, *Mus musculus*, was chosen as a representative of the most commonly observed tetrapod pattern. The lizards were the two *Anolis* species with high TE content in *Hox* clusters (*A. sagrei* and *A. carolinensis*), one *Anolis* species (*A. bartschi*) singled out as having unusually low TE content in *Hox* clusters (Feiner 2016), and one distantly related lizard (the common wall lizards *P. muralis*) with a TE content representative of squamates in general (Fig. 1). In a previous study (Feiner 2016), *A. bartschi* was found to harbor 0.136 TEs per kb in their *Hox* clusters, whereas *A. sagrei* and *A. carolinensis* scored substantially higher TE densities with 0.767 and 0.573, respectively. The three *Anolis* species can be regarded as distantly related with divergence times of 44 million years between *A. sagrei* and *A. carolinensis*, and 47 million years between this species pair and *A. bartschi* (Poe et al. 2017). Expression patterns were highly consistent in location and intensity for a given gene in a particular species, and Figures 4 and 5 show representative results. Expression patterns of *HoxA13*, ‐*B13*, and *‐C13* genes are conserved between *M. musculus* and all lizard species examined (Fig. 4). *HoxA13* is expressed in fore‐ and hindlimb buds and tail tissue, and both *HoxB13* and *‐C13* are expressed in tail tissue only (Fig. 4). In contrast, *HoxD13* is expressed in the fore‐ and hindlimb buds and tail tissue of *M. musculus*, *P. muralis*, and *A. bartschi* (Fig. 5A–5C), but only expressed in the fore‐ and hindlimb buds, not tail tissue, of *A. carolinensis* and *A. sagrei* (Fig. 5D an 5E). The lack of tail‐associated expression of *HoxD13* was confirmed in younger (stages 29 and 30) and older (stages 33 and 34) embryos of *A. carolinensis* and *A. sagrei* (data not shown).

## Discussion

The mechanism of regulation of *Hox* genes suggests that structural changes of *Hox* clusters could cause expression patterns to deviate from the otherwise highly conserved pattern in tetrapods. A candidate for achieving structural changes are transposable elements. TEs can rewire regulatory landscapes (Slotkin and Martienssen 2007; Feschotte 2008; Cowley and Oakey 2013; Sundaram et al. 2014), affect chromatin characteristics (Kidwell and Lisch 1997), and modify distances between genetic elements (e.g., cis‐regulatory elements, promoters, and gene bodies). Squamate genomes are unusually rich and variable in TE contents (Pasquesi et al. 2018) and their *Hox* clusters are enriched in TEs compared to other vertebrate *Hox* clusters (Di‐Poï et al. 2009, 2010; Feiner 2016). Here I addressed the relationship between genomic and *Hox* cluster‐specific TE dynamics to examine evidence for an association between TE invasion, *Hox* cluster expansion, and *Hox* gene expression.

There is generally a linear relationship between the TE contents of *Hox* clusters and whole genomes in lizards and snakes. The TE content of *Hox* clusters is consistently 50% lower than in the genome at large. The exception is found in the extraordinarily species‐rich lizard genus *Anolis* (Losos 2009), with two distantly related species, *A. carolinensis* and *A. sagrei*, exhibiting exceptionally TE‐rich *Hox* clusters. These two species are also characterized by high genome‐wide TE contents. This could imply that genome‐wide expansion of TEs is associated with a failure of the mechanisms that normally restrict TEs from sensitive regions such as *Hox* clusters. As a general rule, this explanation fails given that the Japanese gecko *Gekko japonicus* has an even higher genomic TE content than *A. carolinensis* and *A. sagrei*, but a *Hox* cluster TE content that is less than 50% of genomic TE content. Interestingly, a third species of *Anolis* lizards, *A. auratus*, did not deviate from the relationship between *Hox* cluster and genomic TE contents observed across squamates. Given that TEs in *Anolis Hox* clusters have been shown to accumulate during or after speciation events (Feiner 2016), the low rate of speciation in the recent evolutionary history of *A. auratus*, compared to *A. carolinensis* and *A. sagrei*, might explain this difference. The timing of TE accumulation is crucial to understand the relationship between TE content in contemporary genomes and past speciation. The lifecycle of a given TE is typically a period of high activity, which increases its copy number in the genome, followed by a period of inactivity (often due to silencing in the host genome). Consequently, individual TE copies accumulate mutations and decay over time. Thus, signals of older activity periods, such as those that occurred during early squamate diversification (up to 250 million years ago; Simoes et al. 2018), are either decayed or superseded by more recent TE insertions. Further studies on the relationship between TE activity and rates of speciation in taxa with well‐resolved phylogenies are required to test this hypothesis.

The question of how organisms can protect sensitive regions such as *Hox* clusters from TE insertions remains open. Potential mechanisms involve those that prevent TE accumulation (e.g., preventing insertions through chromatin modifications) or those that remove inserted TEs (e.g., through selective processes). Although genome‐wide data of sufficient quality only exists for three *Anolis* species, it is suggestive that the two species with signatures of comparably recent TE activity (*A. carolinensis* and *A. sagrei*) show a much higher TE content in their *Hox* clusters than the species with older TE activity (*A. auratus*; Figs. 2 and 3 and Fig S5). This could be explained by TE removal from *Hox* clusters that operate with a time lag in respect to genome‐wide TE activity; during periods of high TE activity, TEs proliferate in the genome and are subsequently gradually removed from *Hox* clusters at a higher rate than in the genome at large. Following the positive association between speciation and TE accumulation in *Hox* clusters, I therefore propose the following model: (1) TEs accumulate during speciation events. The signal of this accumulation depends on the age of the process, being evident only at shallow taxonomic levels. This is followed by (2) TE removal from sensitive genomic regions such as *Hox* clusters through selective and neutral processes. The latter process might be modulated by factors such as effective population size, which should positively influence the efficacy of selective TE removal. With increasing genomic resources for more closely related groups of species, both predictions of this model should be testable in the near future.

It has been suggested that the invasion of *Hox* clusters by TEs might explain the dramatic changes in body plans in squamates (Di‐Poï et al. 2010), such as loss of limbs and body elongation. However, there is no TE enrichment in the *Hox* clusters of snakes or snake‐like species (e.g., snakes and the Asian glass lizard, *Ophisaurus gracilis*). Although one cannot exclude the possibility that the invasion of specific TEs in *Hox* clusters has been responsible for phenotypic modifications (perhaps a more likely scenario), my results do not support global structural changes in *Hox* clusters underlying the evolution of highly derived body plans in squamates (see also Feiner 2016).

Nevertheless, expression patterns of one out of four *Hox* genes were found to be aberrant in the two *Anolis* species with elevated levels of *Hox* cluster TE content (*A. carolinensis* and *A. sagrei*). The two species with moderate to low TE content (the European lacertid *P. muralis* and the West Cuban anole *A. bartschi*) showed the typical vertebrate expression patterns (previously established also in a whiptail lizard, family Teiidae; Di‐Poï et al. 2010). Interestingly, the loss of expression in postcaudal tail tissue, reported here for *A. carolinensis* and *A. sagrei HoxD13*, has also been found for the corn snake *HoxA13* and ‐*D13* genes (Di‐Poï et al. 2010). Thus, it appears that the loss of postcaudal expression of *Hox13* genes has evolved repeatedly. The phenotypic effects of this loss may be prevented by redundancy with coexpressed *Hox13* genes. For snakes, it has been suggested that the loss of postcaudal expression of *HoxA13* and ‐*D13* removed signals important for termination of axis elongation, thereby facilitating the elongated body plan of snakes. However, the loss of *HoxD13* expression reported here is not associated with extended axis elongation (neither *A. carolinensis* nor *A. sagrei* has a particularly long tail). Thus, the phenotypic effect of this loss of an expression domain remains unclear.

The proposed model of *Hox* gene regulation in vertebrates emphasizes the structural organization of *Hox* clusters as a causal factor underlying the spatial and temporal collinearity of expression (e.g., Mallo and Alonso 2013). Individual *Hox* genes are activated in a wave‐like manner with Hox13 paralog genes being activated last and in the most posterior embryonic regions. Assuming that the relative location of a given gene in the cluster determines its time and place of expression, an enlarged cluster would primarily affect the expression of the most posterior genes, that is, Hox13 genes. The present finding of disrupted *HoxD13* expression in species with enlarged *Hox* clusters, caused by high TE content, is consistent with this hypothesis. Thus, an impact of TEs on the regulation of the developmentally crucial *Hox* genes is expected from the proposed model of *Hox* gene regulation. More fine‐scale mapping of TE insertion sites relative to gene regulatory elements will help to dissect a potential causal role of TEs on *Hox* gene functions in the future. The recent development of technology for genome manipulation of nonmodel organisms (Rasys et al. 2019) may enable direct tests of the impact of TE‐mediated cluster elongation on *Hox* gene expression patterns, chromatin modifications, and other functional genomic aspects of *Hox* clusters.

## Conclusion

In summary, this study demonstrates that, in contrast to other vertebrates, *Hox* clusters of snakes and lizards can harbor significant amounts of TEs in the *Hox* clusters, reaching 75% of genome‐wide TE content in some *Anolis* lizards. Based on the age of TE invasions, this suggests that genome‐wide bursts of TEs during speciation are followed by selective removal of TEs from *Hox* clusters. The excess of TEs in *Hox* clusters in *Anolis* lizards is associated with changes in gene expression, which is consistent with a role of TEs in development as well as in evolution.

Associate Editor: A. Goswami

## Supporting information


**Table S1**. Information on publicly available genomes used in this study.
**Table S2**. Information of isolated fragments and primer sequences used to obtain *Hox13* genes of four lizards and mouse.

**Figure S1**. Relationship between TE content in *Hox* clusters and whole genomes among squamates using homology‐based TE annotation.

**Figure S2**. TE contents across *Hox* cluster‐containing chromosomal regions.

**Figure S3**. *Anolis carolinensis* and *A. sagrei* TE classes across *Hox* cluster‐containing chromosomal regions.

**Figure S4**. Visualization of pairwise comparison of the four *Hox* clusters between the wall lizard *Podarcis muralis* and the green anole *Anolis carolinensis*.

**Figure S5**. Age of TEs in relation to speciation events in three *Anolis* species.

## References

[evl3131-bib-0001] Bao, Z. , and S. R. Eddy . 2002 Automated de novo identification of repeat sequence families in sequenced genomes. Genome Res. 12:1269–1276. 12176934 10.1101/gr.88502 PMC186642

[evl3131-bib-0002] Cohn, M. J. , and C. Tickle . 1999 Developmental basis of limblessness and axial patterning in snakes. Nature 399:474–479. 10365960 10.1038/20944

[evl3131-bib-0003] Cowley, M. , and R. J. Oakey . 2013 Transposable elements re‐wire and fine‐tune the transcriptome. PLoS Genet. 9:e1003234. 23358118 10.1371/journal.pgen.1003234 PMC3554611

[evl3131-bib-0004] Deschamps, J. , and J. van Nes . 2005 Developmental regulation of the Hox genes during axial morphogenesis in the mouse. Development 132:2931–2942. 15944185 10.1242/dev.01897

[evl3131-bib-0005] Di‐Poï, N. , J. I. Montoya‐Burgos , and D. Duboule . 2009 Atypical relaxation of structural constraints in Hox gene clusters of the green anole lizard. Genome Res. 19:602–610. 19228589 10.1101/gr.087932.108 PMC2665779

[evl3131-bib-0006] Di‐Poï, N. , J. I. Montoya‐Burgos , H. Miller , O. Pourquie , M. C. Milinkovitch , and D. Duboule . 2010 Changes in Hox genes' structure and function during the evolution of the squamate body plan. Nature 464:99–103. 20203609 10.1038/nature08789

[evl3131-bib-0007] Duboule, D. 2007 The rise and fall of Hox gene clusters. Development 134:2549–2560. 17553908 10.1242/dev.001065

[evl3131-bib-0008] Duboule, D. , and P. Dolle . 1989 The structural and functional organization of the murine HOX gene family resembles that of Drosophila homeotic genes. EMBO J. 8:1497–1505. 2569969 10.1002/j.1460-2075.1989.tb03534.x PMC400980

[evl3131-bib-0009] Dufaure, J. , and J. Hubert . 1961 Table de developpement du lezard vivipare: Lacerta vivipara. Arch. d'Anat. Microsc. Morphol. Exp. 50:309–328.

[evl3131-bib-0010] Feiner, N. 2016 Accumulation of transposable elements in Hox gene clusters during adaptive radiation of Anolis lizards. Proc. R. Soc. B Biol. Sci. 283:pii: 20161555. 10.1098/rspb.2016.1555 PMC5069512 27733546

[evl3131-bib-0011] Ferraiuolo, M. A. , M. Rousseau , C. Miyamoto , S. Shenker , X. Q. Wang , M. Nadler , M. Blanchette , and J. Dosti . 2010 The three‐dimensional architecture of Hox cluster silencing. Nucleic Acids Res. 38:7472–7484. 20660483 10.1093/nar/gkq644 PMC2995065

[evl3131-bib-0012] Feschotte, C. 2008 Transposable elements and the evolution of regulatory networks. Nat. Rev. Genet. 9:397–405. 18368054 10.1038/nrg2337 PMC2596197

[evl3131-bib-0013] Gaunt, S. J. 1994 Conservation in the Hox code during morphological evolution. Int. J. Dev. Biol. 38:549–552. 7848839

[evl3131-bib-0014] Graham, A. , N. Papalopulu , and R. Krumlauf . 1989 The murine and Drosophila homeobox gene complexes have common features of organization and expression. Cell 57:367–378. 2566383 10.1016/0092-8674(89)90912-4

[evl3131-bib-0015] Head, J. J. , and P. D. Polly . 2015 Evolution of the snake body form reveals homoplasy in amniote Hox gene function. Nature 520:86–89. 25539083 10.1038/nature14042

[evl3131-bib-0016] Holland, P. W. , J. Garcia‐Fernandez , N. A. Williams , and A. Sidow . 1994 Gene duplications and the origins of vertebrate development. Dev. Suppl. 125–133. 7579513

[evl3131-bib-0017] Hubbard, T. J. , B. L. Aken , S. Ayling , B. Ballester , K. Beal , E. Bragin , S. Brent , Y. Chen , P. Clapham , L. Clarke , et al. 2009 Ensembl 2009. Nucleic Acids Res. 37:D690–D697. 19033362 10.1093/nar/gkn828 PMC2686571

[evl3131-bib-0018] Jurka, J. 2000 Repbase update: A database and an electronic journal of repetitive elements. Trends Genet. 16:418–420. 10973072 10.1016/s0168-9525(00)02093-x

[evl3131-bib-0019] Kidwell, M. G. , and D. Lisch . 1997 Transposable elements as sources of variation in animals and plants. Proc. Natl. Acad. Sci. USA 94:7704–7711. 9223252 10.1073/pnas.94.15.7704 PMC33680

[evl3131-bib-0020] Kmita, M. , and D. Duboule . 2003 Organizing axes in time and space; 25 years of colinear tinkering. Science 301:331‐333. 12869751 10.1126/science.1085753

[evl3131-bib-0021] Lemons, D. , and W. McGinnis . 2006 Genomic evolution of Hox gene clusters. Science 313:1918–1922. 17008523 10.1126/science.1132040

[evl3131-bib-0022] Losos, J. B. 2009 Lizards in an evolutionary tree: Ecology and adaptive radiation of anoles. Univ. of California Press, Berkeley, CA.

[evl3131-bib-0023] Mallo, M. , and C. R. Alonso . 2013 The regulation of Hox gene expression during animal development. Development 140:3951–3963. 24046316 10.1242/dev.068346

[evl3131-bib-0024] Papageorgiou, S. 2017 Physical forces may cause the HoxD gene cluster elongation. Biology 6 10.3390/biology6030032. PMC5617920 28644379

[evl3131-bib-0025] Pasquesi, G. I. M. , R. H. Adams , D. C. Card , D. R. Schield , A. B. Corbin , B. W. Perry , J. Reyes‐Velasco , R. P. Ruggiero , M. W. Vandewege , J. A. Shortt , et al. 2018 Squamate reptiles challenge paradigms of genomic repeat element evolution set by birds and mammals. Nat. Commun. 9:2774. 30018307 10.1038/s41467-018-05279-1 PMC6050309

[evl3131-bib-0026] Poe, S. , A. Nieto‐Montes de Oca , O. Torres‐Carvajal , K. De Queiroz , J. A. Velasco , B. Truett , L. N. Gray , M. J. Ryan , G. Köhler , F. Ayala‐Varela , et al. 2017 A phylogenetic, biogeographic, and taxonomic study of all extant species of Anolis (Squamata; Iguanidae). Syst. Biol. 66:663‐697. 28334227 10.1093/sysbio/syx029

[evl3131-bib-0027] Price, A. L. , N. C. Jones , and P. A. Pevzner . 2005 De novo identification of repeat families in large genomes. Bioinformatics 21:i351–i358. 15961478 10.1093/bioinformatics/bti1018

[evl3131-bib-0028] Quinlan, A. R. , and I. M. Hall . 2010 BEDTools: A flexible suite of utilities for comparing genomic features. Bioinformatics 26:841–842. 20110278 10.1093/bioinformatics/btq033 PMC2832824

[evl3131-bib-0029] Rasys, A. M. , S. Park , R. E. Ball , A. J. Alcala , J. D. Lauderdale , and D. B. Menke . 2019 CRISPR‐Cas9 gene editing in lizards through microinjection of unfertilized oocytes. bioRxiv:591446. 10.1016/j.celrep.2019.07.089 PMC6727204 31461646

[evl3131-bib-0030] Sanger, T. J. , J. B. Losos , and J. J. Gibson‐Brown . 2008 A developmental staging series for the lizard genus Anolis: A new system for the integration of evolution, development, and ecology. J. Morphol. 269:129–137. 17724661 10.1002/jmor.10563

[evl3131-bib-0031] Simoes, T. R. , M. W. Caldwell , M. Talanda , M. Bernardi , A. Palci , O. Vernygora , F. Bernardini , L. Mancini , and R. L. Nydam . 2018 The origin of squamates revealed by a Middle Triassic lizard from the Italian Alps. Nature 557:706–709. 29849156 10.1038/s41586-018-0093-3

[evl3131-bib-0032] Slotkin, R. K. , and R. Martienssen . 2007 Transposable elements and the epigenetic regulation of the genome. Nat. Rev. Genet. 8:272–285. 17363976 10.1038/nrg2072

[evl3131-bib-0033] Smit, A. F. A. , and R. Hubley . RepeatModeler Open‐1.0. 2008.

[evl3131-bib-0034] Smit, A. F. A. , R. Hubley , and P. Green . 2013 Repeatmasker Open‐4.0. Available via http://www.repeatmasker.org.

[evl3131-bib-0035] Sundaram, V. , Y. Cheng , Z. Ma , D. Li , X. Xing , P. Edge , M. P. Snyder , and T. Wang . 2014 Widespread contribution of transposable elements to the innovation of gene regulatory networks. Genome Res. 24:1963–1976. 25319995 10.1101/gr.168872.113 PMC4248313

[evl3131-bib-0036] Tarchini, B. , and D. Duboule . 2006 Control of Hoxd genes' collinearity during early limb development. Dev. Cell. 10:93–103. 16399081 10.1016/j.devcel.2005.11.014

[evl3131-bib-0037] Tollis, M. , E. D. Hutchins , J. Stapley , S. M. Rupp , W. L. Eckalbar , I. Maayan , E. Lasku , C. R. Infante , S. R. Dennis , J. A. Robertson , et al. 2018 Comparative genomics reveals accelerated evolution in conserved pathways during the diversification of Anole lizards. Genome Biol. Evol. 10:489–506. 29360978 10.1093/gbe/evy013 PMC5798147

[evl3131-bib-0038] Wakasa, H. , A. Cadiz , L. M. Echenique‐Diaz , W. M. Iwasaki , N. Kamiyama , Y. Nishimura , H. Yokoyama , K. Tamura , and M. Kawata . 2015 Developmental stages for the divergence of relative limb length between a twig and a trunk‐ground Anolis lizard species. J. Exp. Zool. 324:410–423. 10.1002/jez.b.22627 26055630

[evl3131-bib-0039] Woltering, J. M. 2012 From lizard to snake; behind the evolution of an extreme body plan. Curr. Genomics 13:289–299. 23204918 10.2174/138920212800793302 PMC3394116

[evl3131-bib-0040] Woltering, J. M. , F. J. Vonk , H. Muller , N. Bardine , I. L. Tuduce , M. A. de Bakker , W. Knöchel , I. O. Sirbu , A. J. Durston , and M. K. Richardson . 2009 Axial patterning in snakes and caecilians: Evidence for an alternative interpretation of the Hox code. Dev. Biol. 332:82–89. 19409887 10.1016/j.ydbio.2009.04.031

