# Supplementary material for: Evolutionary lability in Hox cluster structure and gene expression in Anolis lizards
Source: Evol Lett. 2019 Aug 6;3(5):474–84. doi: 10.1002/evl3.131 (PMC6791295; doi:10.1002/evl3.131)
Supplement: Supplementary file 1 — Table S1. Information on publicly available genomes used in this study. Table S2. Information of isolated fragments and primer sequences used to obtain Hox13 genes of four lizards and mouse. [file EVL3-3-474-s001.pdf]

Electronic supplementary material

Evolutionary lability in *Hox* cluster structure and gene expression in *Anolis* lizards

Nathalie Feiner[1,2]

[1]Department of Biology, Lund University, Sölvegatan 37, 223 62 Lund, Sweden

[2]E-mail: nathalie.feiner@biol.lu.se

Supplementary figures

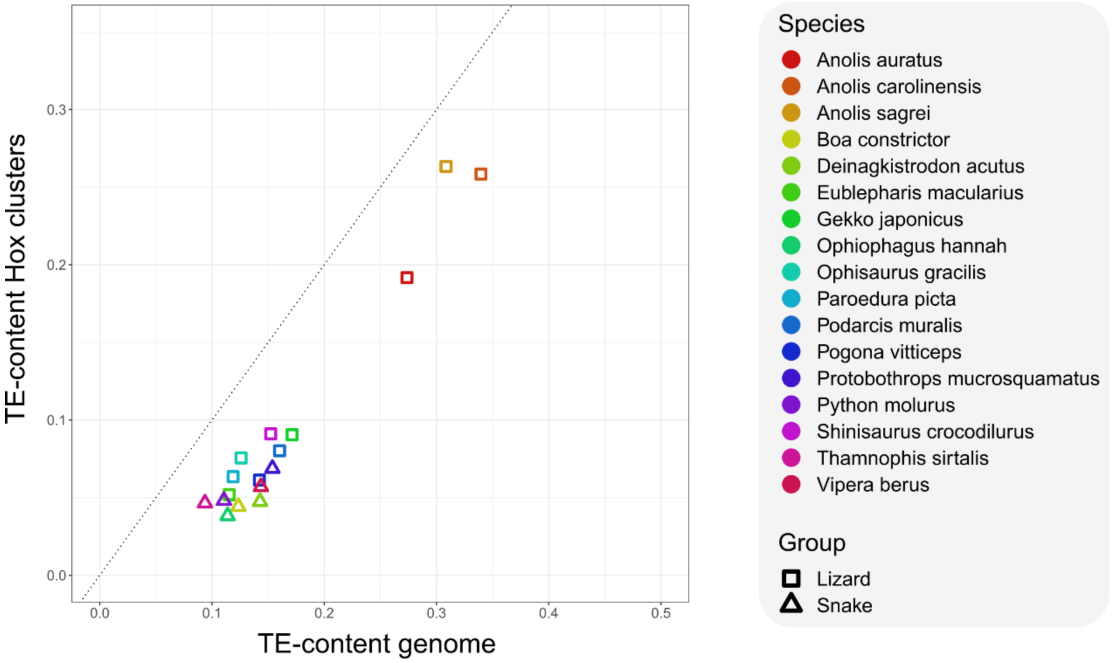

Figure S1. Relationship between TE-content in *Hox* clusters and whole genomes amongst squamates using homology-based TE annotation. Plot shows the relationship of *Hox* cluster versus genome-wide TE-content, analogous to Fig. 2, but with homology-based TE annotation (based on vertebrate TEs) instead of *de novo* TE prediction. Estimation of TE-contents is biased towards *Anolis* species because the ‘vertebrate’ TE library is over-represented by entries of the species *Anolis carolinensis* (371 entries of a total of 406 entries for lepidosauria).

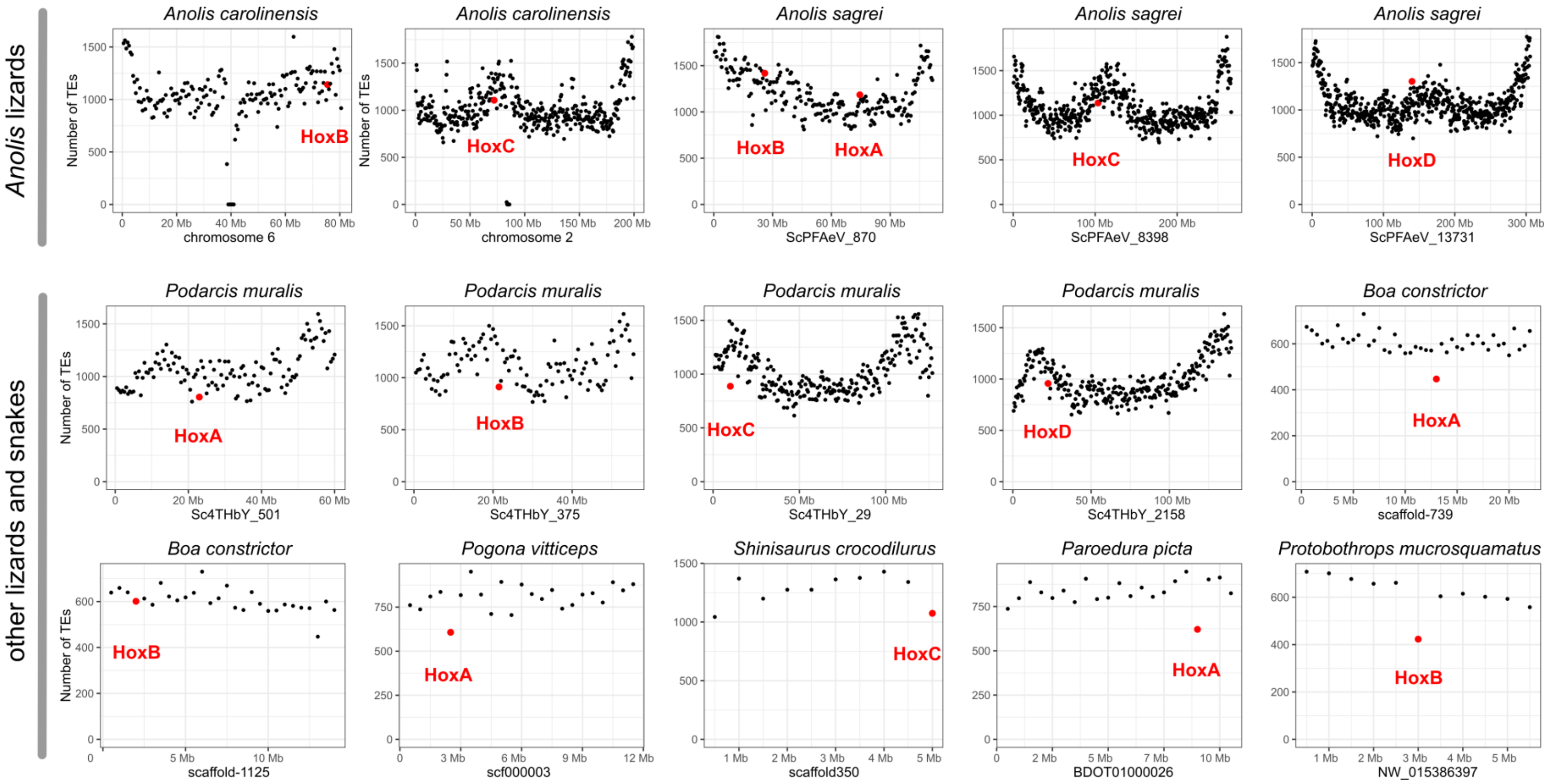

Figure S2. TE-contents across *Hox* cluster-containing chromosomal regions. Plots show TE-contents in 500 kb bins along *Hox* cluster-containing chromosomal regions. Each dot represents the number of TEs for a 500 kb region. Black dots mark regions outside the *Hox* clusters, and red dots mark *Hox* cluster-containing chromosomal regions. Plots are only shown for *Hox* clusters that are located on scaffolds that are at least

5 Mbs (10 bins) in size. *Hox* cluster TE content is generally lower than neighbouring regions for non-*Anolis* squamates (middle and lowest row), while *Anolis Hox* cluster-containing regions are equal or higher in TE content compared to neighbouring chromosomal regions (upper row).

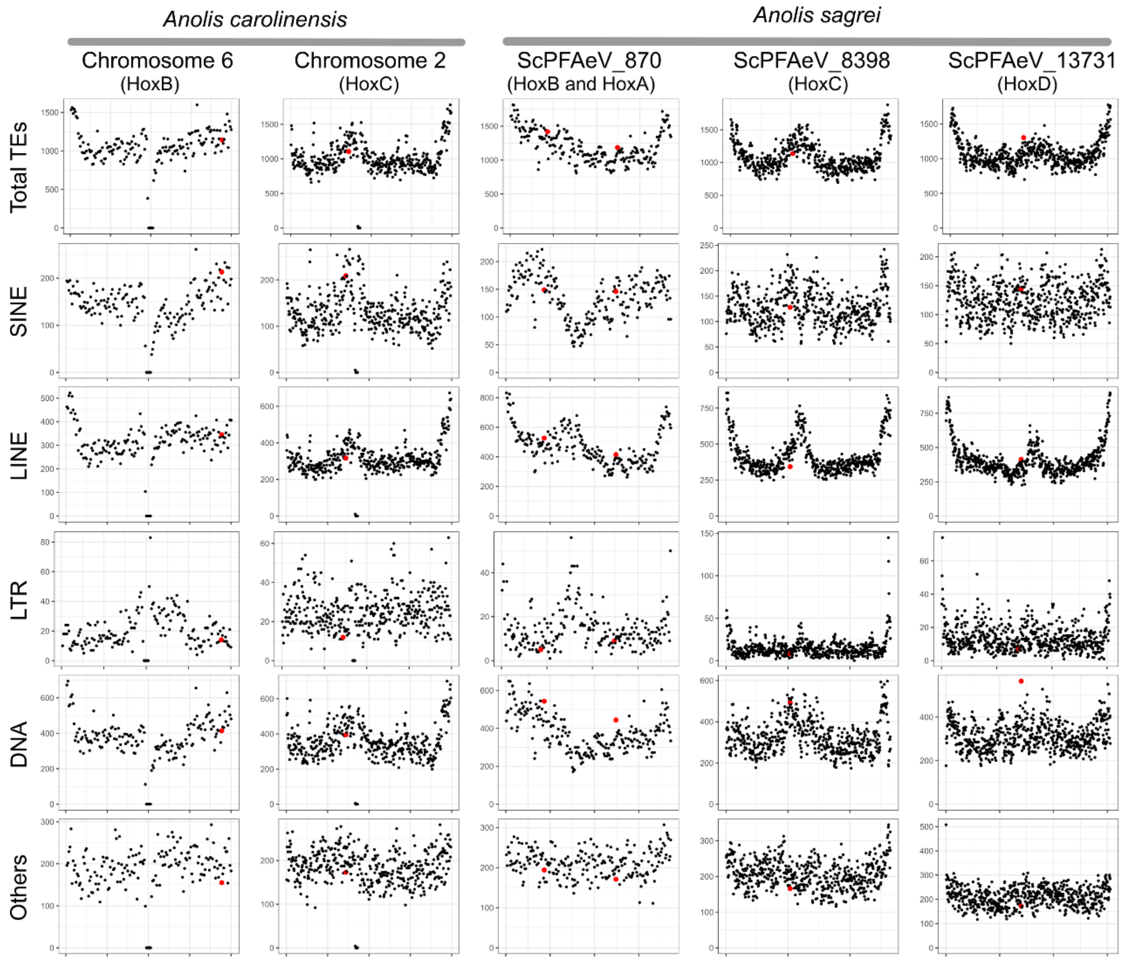

Figure S3. *Anolis carolinensis* and *A. sagrei* TE classes across *Hox* cluster-containing chromosomal regions. Plots show number of individual elements per class in 500 kb bins along *Hox* cluster-containing chromosomal regions for the two *Anolis* lizards with high *Hox* cluster TE-content. Black dots mark regions outside the *Hox* clusters, and red dots mark *Hox* cluster-containing chromosomal regions. Top row shows total amounts of TEs (similar to supplementary figure S3). Chromosome-wide pattern of TE class distribution reveals class-specific patterns, e.g. LINE enrichment in centromeric and telomeric regions, while SINEs show the opposite pattern. Overall, SINE elements in *A. carolinensis*, and DNA transposons in *A. sagrei*, contribute most to the relative TE-rich nature of *Hox* clusters in these two species.

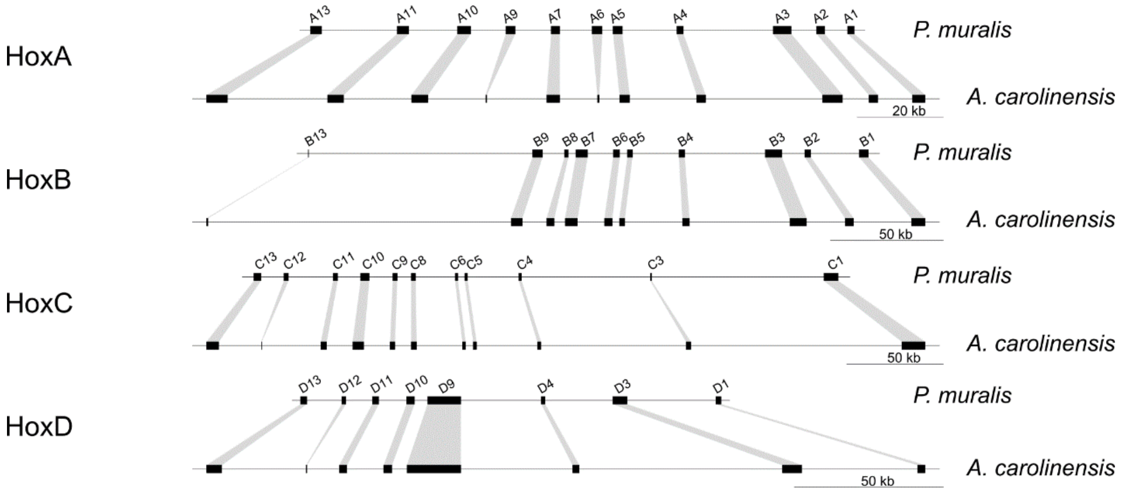

Figure S4. Visualization of pairwise comparison of the four *Hox* clusters between the wall lizard *Podarcis muralis* and the green anole *Anolis carolinensis*. Gene bodies (coding sequence plus introns) are shown as black boxes and intergenic regions as grey lines. Shaded areas denote orthologous relationship between *Hox* genes. The comparison illustrates that the gene body size is largely constant between the two species, whereas intergenic regions are elongated in *A. carolinensis*, and this elongation is evenly distributed across all clusters.

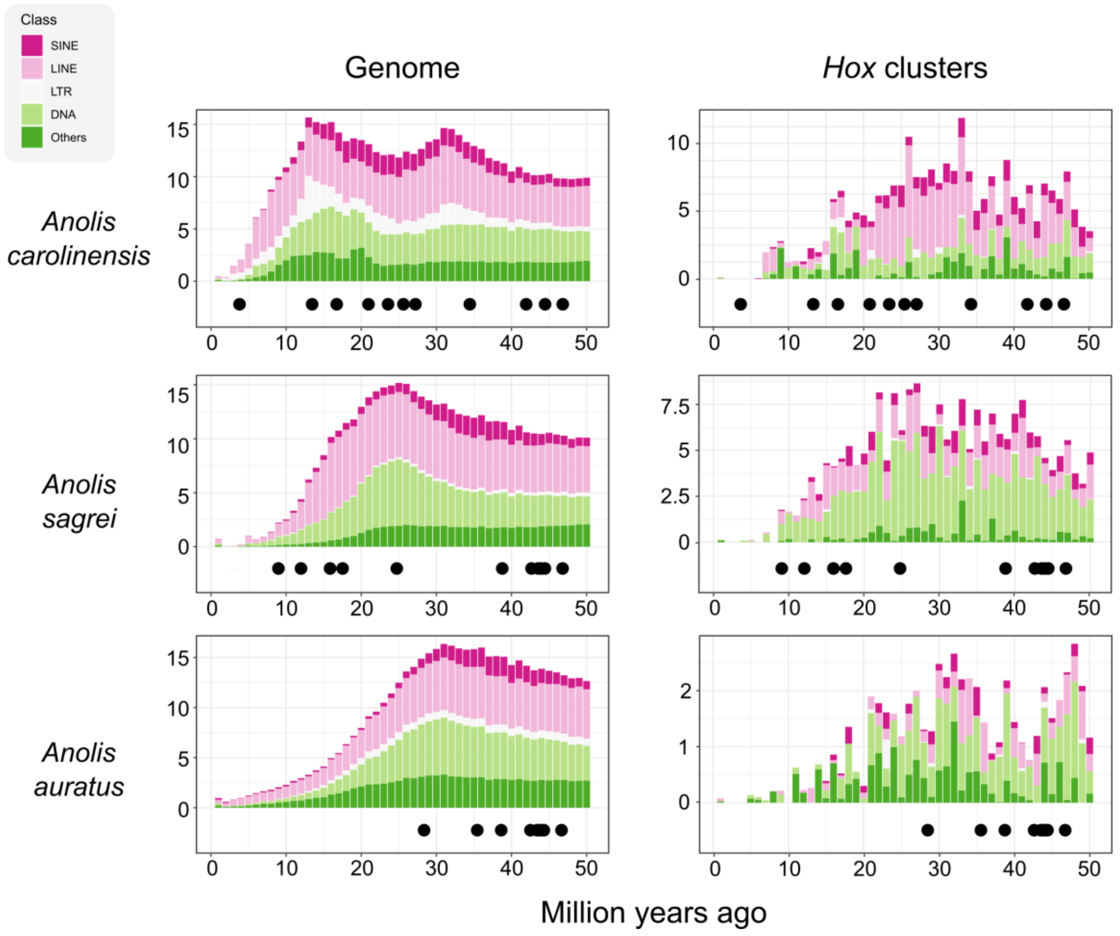

Figure S5. Age of TEs in relation to speciation events in three *Anolis* species. The age of individual TEs was estimated based on their divergence from the consensus for each TE family and calibrated with an estimated substitution rate of the genus *Anolis* (Tollis et al. 2018). Frequencies are given in millions for genomic TEs, and in thousands for *Hox* cluster TEs. Below the frequency distribution plots, the estimated speciation events (Poe et al. 2017) are indicated for each species by black circles. The last common ancestor of *A. carolinensis*, *A. sagrei* and *A. auratus* was estimated to date back to 46.77 million years ago (Poe et al. 2017).

Supplementary tables

Table S1. Information on publicly available genomes used in this study.

Latin species name	English common name	Reference	URL source for download (file name)	Genome size in gb*	N50	Complete Hox clusters (length in kb)
Anolis carolinensis	green anole	(Alfoldi et al. 2011)	†	NA / 1.78	150.6 Mb	HoxA (170) HoxB (326) HoxC (379) HoxD (243)
A. apletophallus	slender anole	(Tollis et al. 2018)	https://dataverse.harvard.edu/dataset.xhtml?persistentId=doi:10.7910/DVN/NGSGCG (Aapl1.0.fasta.softmasked.gz)	NA / 2.02	9.5 kb	-
A. auratus	grass anole	(Tollis et al. 2018)	https://dataverse.harvard.edu/dataset.xhtml?persistentId=doi:10.7910/DVN/NGSGCG (Aaur1.0.fa.softmasked.gz)	NA / 2.18	49 kb	HoxA (167) HoxD (176)
A. frenatus	Central American giant anole	(Tollis et al. 2018)	https://dataverse.harvard.edu/dataset.xhtml?persistentId=doi:10.7910/DVN/NGSGCG (Afre1.0.fasta.softmasked.gz)	NA / 2.03	37 kb	-
A. sagrei	brown anole	NA	[unpublished data]	NA / 1.60	232 Mb	HoxA (152) HoxB (345) HoxC (333) HoxD (174)
Eublepharis macularius	Leopard gecko	(Xiong et al. 2016)	http://gigadb.org/dataset/view/id/100246/File_page/3 (E_macularius.scafSeq.fill.FG.gz)	2.23 / 2.02	664 kb	HoxA (114) HoxB (260) HoxC (292) HoxD (136)

Gekko japonicus	Schlegel's Japanese Gecko	(Liu et al. 2015)	ftp://ftp.ncbi.nlm.nih.gov/genomes/all/GCA/001/447/785/GCA_001447785.1_Gekko_japonicus_V1.1 (GCA_001447785.1_Gekko_japonicus_V1.1_genomic.fna.gz)	NA / 2.55	680 kb	-
Ophisaurus gracilis	Asian glass lizard	(Song et al. 2015)	http://gigadb.org/dataset/100119 (O.gracilis.final.assembly.fa)	1.71 / 1.78	1.27 Mb	HoxB (287) HoxD (145)
Paroedura picta	Madagascar ground gecko	(Hara et al. 2018)	https://www.ncbi.nlm.nih.gov/Traces/wgs/?display=contigs&page=1	1.80 / 1.69	4.1 Mb	HoxA (113) HoxB (263)
Podarcis muralis	common wall lizard	(Andrade et al. 2018)	https://www.ncbi.nlm.nih.gov/genome/?term=%22Podarcis+muralis%22	NA / 1.5	95 Mb	HoxA (124) HoxB (251) HoxC (305) HoxD (143)
Pogona vitticeps	bearded dragon	(Georges et al. 2015)	https://www.ncbi.nlm.nih.gov/genome/?term=Pogona+vitticeps (GCF_900067755.1_pvi1.1_genomic.fna.gz)	NA / 1.77	2.29 Mb	HoxA (143) HoxB (277) HoxC (304) HoxD (153)
Shinisaurus crocodilurus	Chinese crocodile lizard	(Gao et al. 2017)	http://gigadb.org/dataset/view/id/100315/File_page/4 (Shinisaurus_crocodilurus.fa.gz)	NA / 2.24	1.47 Mb	HoxA (128) HoxC (347)
Boa constrictor constrictor	boa constrictor	(Bradnam et al. 2013)	http://gigadb.org/dataset/view/id/100060/File_page/9 (snake_7C_scaffolds.fa.gz)	1.6/1.44	3.80 Mb	HoxA (123) HoxB (249) HoxD (126)
Crotalus horridus	timber rattlesnake	NA	https://www.ncbi.nlm.nih.gov/genome/16679?genome_assembly_id=274149	NA/1.52	5.8 kb	-

Crotalus mitchelli pyrrhus	speckled rattlesnake	(Gilbert et al. 2014)	https://www.ncbi.nlm.nih.gov/genome/56045?genome_assembly_id=325142 (GCA_000737285.1_CrotMitch1.0_genomic.fna.gz)	NA/1.13	5.3 kb	-
Deinagkistrodon acutus	five-pacer viper	(Yin et al. 2016)	http://gigadb.org/dataset/100196 (Deinagkistrodon_acutus.fa.gz)	1.43 / 1.47	2.12 Mb	HoxA (122) HoxB (249) HoxD (135)
Ophiophagus hannah	king cobra	(Vonk et al. 2013)	https://www.ncbi.nlm.nih.gov/genome/?term=Ophiophagus+hanna (GCA_000516915.1_OphHan1.0_genomic.fna.gz)	NA / 1.66	226 kb	HoxA (121) HoxB (237) HoxD (138)
Pantherophis guttatus	corn snake	(Ullate-Agote et al. 2014)	https://www.ncbi.nlm.nih.gov/genome/40371?genome_assembly_id=249454	NA/1.40	2.4 kb	-
Protobothrops mucrosquamatus	Brown spotted pit viper	(Aird et al. 2017)	https://www.ncbi.nlm.nih.gov/genome/18192?genome_assembly_id=280649	NA/1.67	21.9 kb	HoxA (122) HoxB (231) HoxD (143)
Python molurus	Burmese python	(Castoe et al. 2013)	https://www.ncbi.nlm.nih.gov/genome/17893?genome_assembly_id=48432 (GCF_000186305.1_Python_molurus_bivittatus-5.0.2_genomic.fna.gz)	NA / 1.44	207.5 kb	-
Thamnophis sirtalis	garter snake	(Perry et al. 2018)	https://www.ncbi.nlm.nih.gov/genome/16688?genome_assembly_id=245767 (GCF_001077635.1_Thamnophis_sirtalis-6.0_genomic.fna.gz)	NA / 1.42	10.4 kb	HoxA (127) HoxB (237)
Vipera berus	European viper	NA	https://www.ncbi.nlm.nih.gov/genome/14467?genome_assembly_id=214193	NA/1.53	11.7 kb	HoxA (121) HoxB (235) HoxD (138)

*Genome sizes are given as estimation (either k/mer based or flow cytometry) / actual assembly size

†based on AnoCar2.0

Table S2. Information of isolated fragments and primer sequences used to obtain *Hox13* genes of four lizards and mouse.

Species	Gene	Forward primer	Reverse primer	Method
A. bartschi	*HoxA13*	GAGGAGATCAACAAGAAC	TAATACGACTCACTATAGGGATTGTGACTTGCCTCTCT	[a]
A. bartschi	*HoxB13*	TACTATTCCTACCGCGTG	TAATACGACTCACTATAGGGCTGGAACCAGATGGTGAT	[a]
A. bartschi	*HoxC13*	ACTTCCCTGATCTTGCAT	TAATACGACTCACTATAGGGGCTTGTTTGTTCTGGCTT	[a]
A. bartschi	*HoxD13*	CAAGGAGGTCTCCTTCTA	TAATACGACTCACTATAGGGAAGCAGAAGGAGAGGCTT	[a]
A. carolinensis	*HoxA13*	TGGAGGAGATCAACAAGA	CCACGCGTCGACTAGTACTT	[b]
A. carolinensis	*HoxB13*	GAGACTTTGCAAAGGAGA	CCACGCGTCGACTAGTACTT	[b]
A. carolinensis	*HoxC13*	ACTTGCAGCAGAAGCCTT	CCACGCGTCGACTAGTACTT	[b]
A. carolinensis	*HoxD13*	CAAGGAGGTCTCCTTCTA	CCACGCGTCGACTAGTACTT	[b]
A. sagrei	*HoxA13*	TGGAGGAGATCAACAAGA	TAATACGACTCACTATAGGGATTGTGACTTGCCTCTCT	[a]
A. sagrei	*HoxB13*	CGACGCGTGAGTTCAGC	GTTCTGGAACCAGATGGTGAT	[c]
A. sagrei	*HoxC13*	ACTTCCCTGATCTTGCAT	TAATACGACTCACTATAGGGGCTTGTTTGTTCTGGCTT	[a]
A. sagrei	*HoxD13*	CAAGGAGGTCTCCTTCTA	CCACGCGTCGACTAGTACTT	[b]
M. musculus	*HoxA13*	AAAGTTGGAGAGTACGGC	TAATACGACTCACTATAGGGTAGCTGGAAATACACTGG	[a]
M. musculus	*HoxB13*	ACCTAAGACAGCGAAGGT	TAATACGACTCACTATAGGGTCTAGAAACTCAAGTGGC	[a]
M. musculus	*HoxC13*	TCTGGCAAGTGGAGTTTT	TTGGAGCAGTACACCTGA	[b]
M. musculus	*HoxD13*	AACTGTCCGAATATGGAG	TAATACGACTCACTATAGGGATAAGGTTCACTGCAAGC	[a]
P. muralis	*HoxA13*	AAAGAAGAGAGTGCCTTACA	TAATACGACTCACTATAGGGGTGTTTACCTGAGCAACC	[a]
P. muralis	*HoxB13*	AAGAAGAGGATCCCTTACA	TAATACGACTCACTATAGGGTAAAGGAGAGAGGCTAGG	[a]
P. muralis	*HoxC13*	CTTTTGGAGGCAGCTACTA	TAATACGACTCACTATAGGGTTCAAAGAGTTCGAATGC	[a]
P. muralis	*HoxD13*	TAAACCAGCCGGATATGT	TAATACGACTCACTATAGGGAAGGTCAGAGGTCAGTGG	[a]

[a] Probe synthesis performed directly using PCR product as template in *in vitro* transcription (reverse primer contains T7 promoter).

[b] First identification of fragment using 3'-RACE. PCR product was cloned using the pGEM®-T Easy Vector System, and plasmids were used as template in *in vitro* transcription.

[c] First identification of fragment using 5'-RACE. PCR product was cloned using the pGEM®-T Easy Vector System, and plasmids were used as template in *in vitro* transcription.

Literature cited

Aird, S. D., J. Arora, A. Barua, L. Qiu, K. Terada, and A. S. Mikheyev. 2017. Population Genomic Analysis of a Pitviper Reveals Microevolutionary Forces Underlying Venom Chemistry. Genome biology and evolution 9:2640-2649.

Alfoldi, J., F. Di Palma, M. Grabherr, C. Williams, L. S. Kong, E. Mauceli, *et al.* 2011. The genome of the green anole lizard and a comparative analysis with birds and mammals. Nature 477:587-591.

Andrade, P., C. Pinho, G. Perez i de Lanuza, S. Afonso, J. Brejcha, C.-J. Rubin, *et al.* 2018. Regulatory changes in pterin and carotenoid genes underlie balanced color polymorphisms in the wall lizard. bioRxiv:481895.

Bradnam, K. R., J. N. Fass, A. Alexandrov, P. Baranay, M. Bechner, I. Birol, *et al.* 2013. Assemblathon 2: evaluating de novo methods of genome assembly in three vertebrate species. GigaScience 2.

Castoe, T. A., A. P. de Koning, K. T. Hall, D. C. Card, D. R. Schield, M. K. Fujita, *et al.* 2013. The Burmese python genome reveals the molecular basis for extreme adaptation in snakes. Proceedings of the National Academy of Sciences of the United States of America 110:20645-20650.

Gao, J., Q. Li, Z. Wang, Y. Zhou, P. Martelli, F. Li, *et al.* 2017. Sequencing, de novo assembling, and annotating the genome of the endangered Chinese crocodile lizard Shinisaurus crocodilurus. GigaScience 6:1-6.

Georges, A., Q. Li, J. Lian, D. O'Meally, J. Deakin, Z. Wang, *et al.* 2015. High-coverage sequencing and annotated assembly of the genome of the Australian dragon lizard Pogona vitticeps. GigaScience 4:45.

Gilbert, C., J. M. Meik, D. Dashevsky, D. C. Card, T. A. Castoe, and S. Schaack. 2014. Endogenous hepadnaviruses, bornaviruses and circoviruses in snakes. Proceedings. Biological sciences / The Royal Society 281:20141122.

Hara, Y., M. Takeuchi, Y. Kageyama, K. Tatsumi, M. Hibi, H. Kiyonari, *et al.* 2018. Madagascar ground gecko genome analysis characterizes asymmetric fates of duplicated genes. BMC biology 16:40.

Liu, Y., Q. Zhou, Y. J. Wang, L. H. Luo, J. Yang, L. F. Yang, *et al.* 2015. Gekko japonicus genome reveals evolution of adhesive toe pads and tail regeneration. Nature communications 6.

Perry, B. W., D. C. Card, J. W. McGlothlin, G. I. M. Pasquesi, R. H. Adams, D. R. Schield, *et al.* 2018. Molecular adaptations for sensing and securing prey and insight into amniote genome diversity from the garter snake genome. Genome biology and evolution.

Poe, S., A. Nieto-Montes de Oca, O. Torres-Carvajal, K. De Queiroz, J. A. Velasco, B. Truett, *et al.* 2017. A Phylogenetic, Biogeographic, and Taxonomic study of all Extant Species of Anolis (Squamata; Iguanidae). Systematic biology 66:663-697.

Song, B., S. Cheng, Y. Sun, X. Zhong, J. Jin, R. Guan, *et al.* 2015. A genome draft of the legless anguid lizard, Ophisaurus gracilis. GigaScience 4:17.

Tollis, M., E. D. Hutchins, J. Stapley, S. M. Rupp, W. L. Eckalbar, I. Maayan, *et al.* 2018. Comparative Genomics Reveals Accelerated Evolution in Conserved Pathways during the Diversification of Anole Lizards. Genome biology and evolution.

Ullate-Agote, A., M. C. Milinkovitch, and A. C. Tzika. 2014. The genome sequence of the corn snake (Pantherophis guttatus), a valuable resource for EvoDevo studies in squamates. The International journal of developmental biology 58:881-888.

Vonk, F. J., N. R. Casewell, C. V. Henkel, A. M. Heimberg, H. J. Jansen, R. J. McCleary, *et al.* 2013. The king cobra genome reveals dynamic gene evolution and adaptation in the snake venom system. Proceedings of the National Academy of Sciences of the United States of America 110:20651-20656.

Xiong, Z., F. Li, Q. Li, L. Zhou, T. Gamble, J. Zheng, *et al.* 2016. Draft genome of the leopard gecko, Eublepharis macularius. GigaScience 5:47.
Yin, W., Z. J. Wang, Q. Y. Li, J. M. Lian, Y. Zhou, B. Z. Lu, *et al.* 2016. Evolutionary trajectories of snake genes and genomes revealed by comparative analyses of five-pacer viper. Nature communications 7:13107.
