# Supplementary figures and images for: Evolutionary lability in Hox cluster structure and gene expression in Anolis lizards
Source: Evol Lett. 2019 Aug 6;3(5):474–84. doi: 10.1002/evl3.131 (PMC6791295; doi:10.1002/evl3.131)

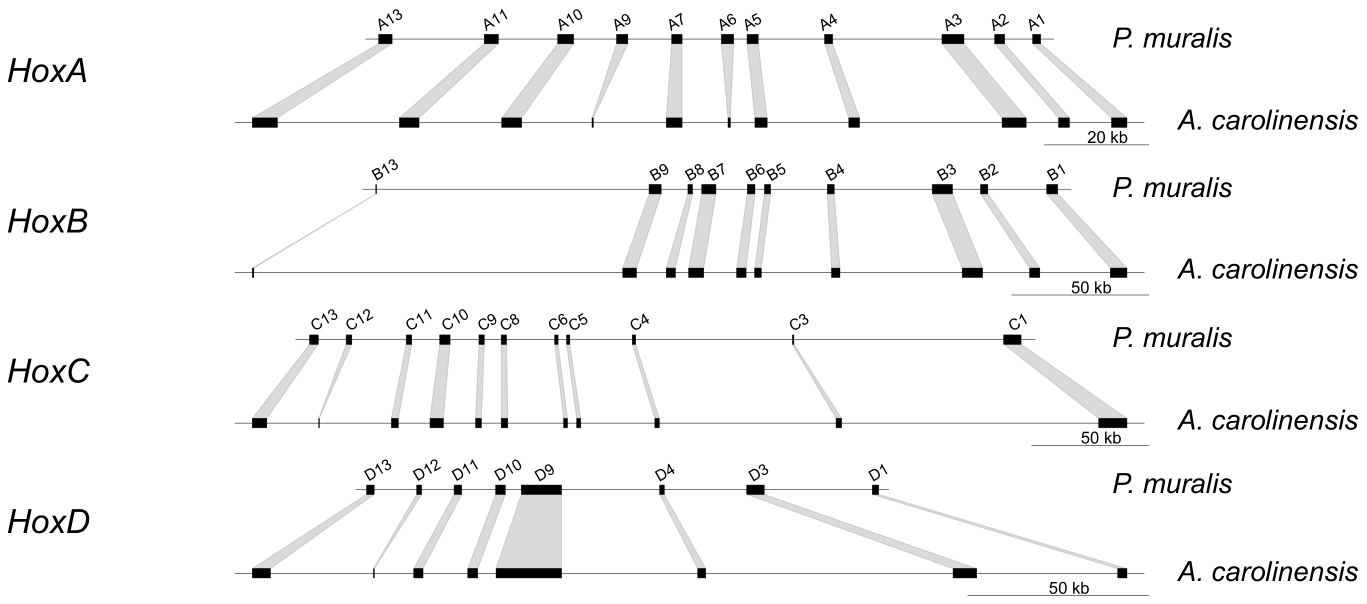

Supplement: Supplementary file 2 — Figure S1. Relationship between TE content in Hox clusters and whole genomes among squamates using homology‐based TE annotation. [file EVL3-3-474-s002.tiff]

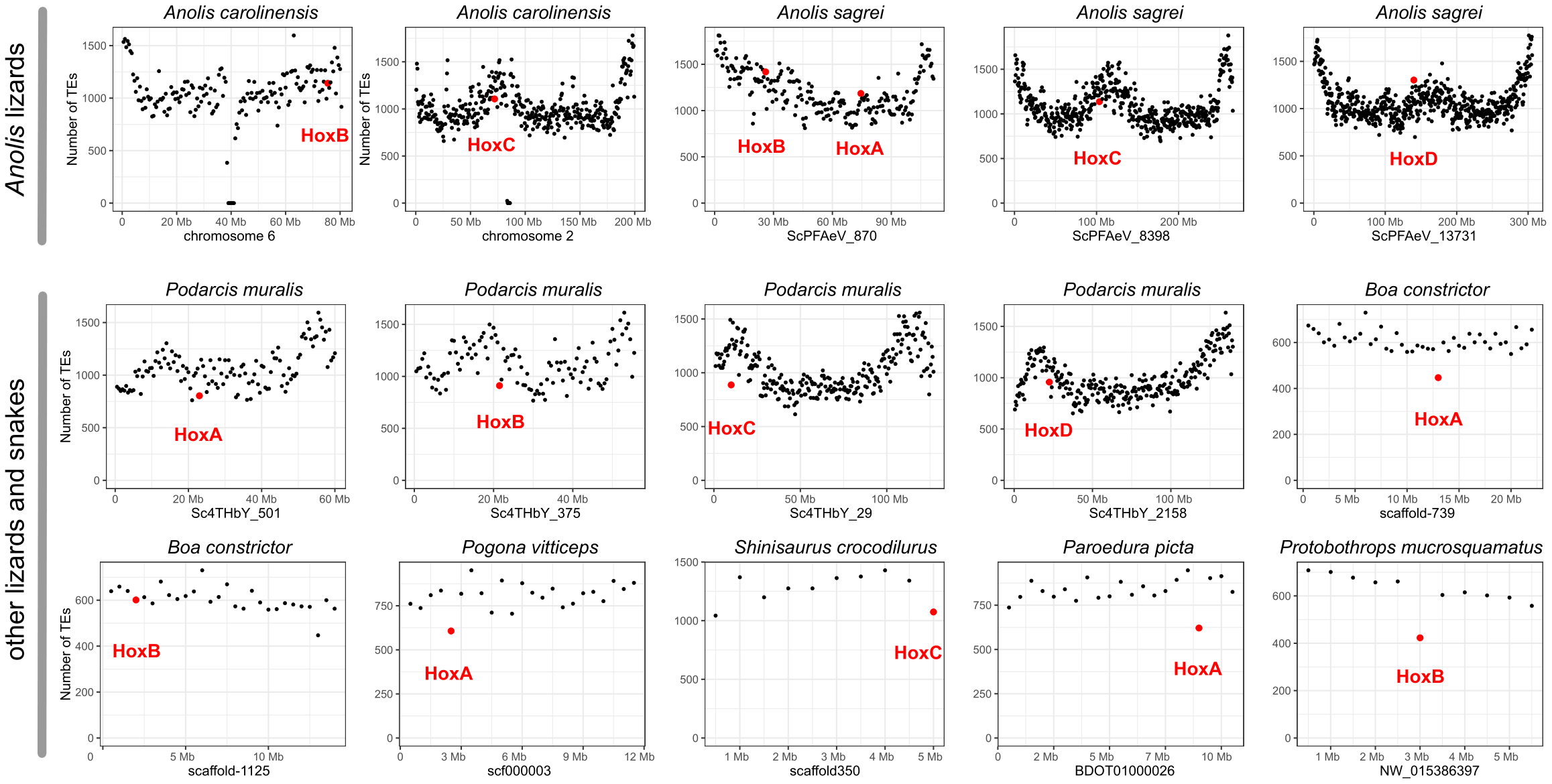

Supplement: Supplementary file 3 — Figure S2. TE contents across Hox cluster‐containing chromosomal regions. [file EVL3-3-474-s003.tiff]

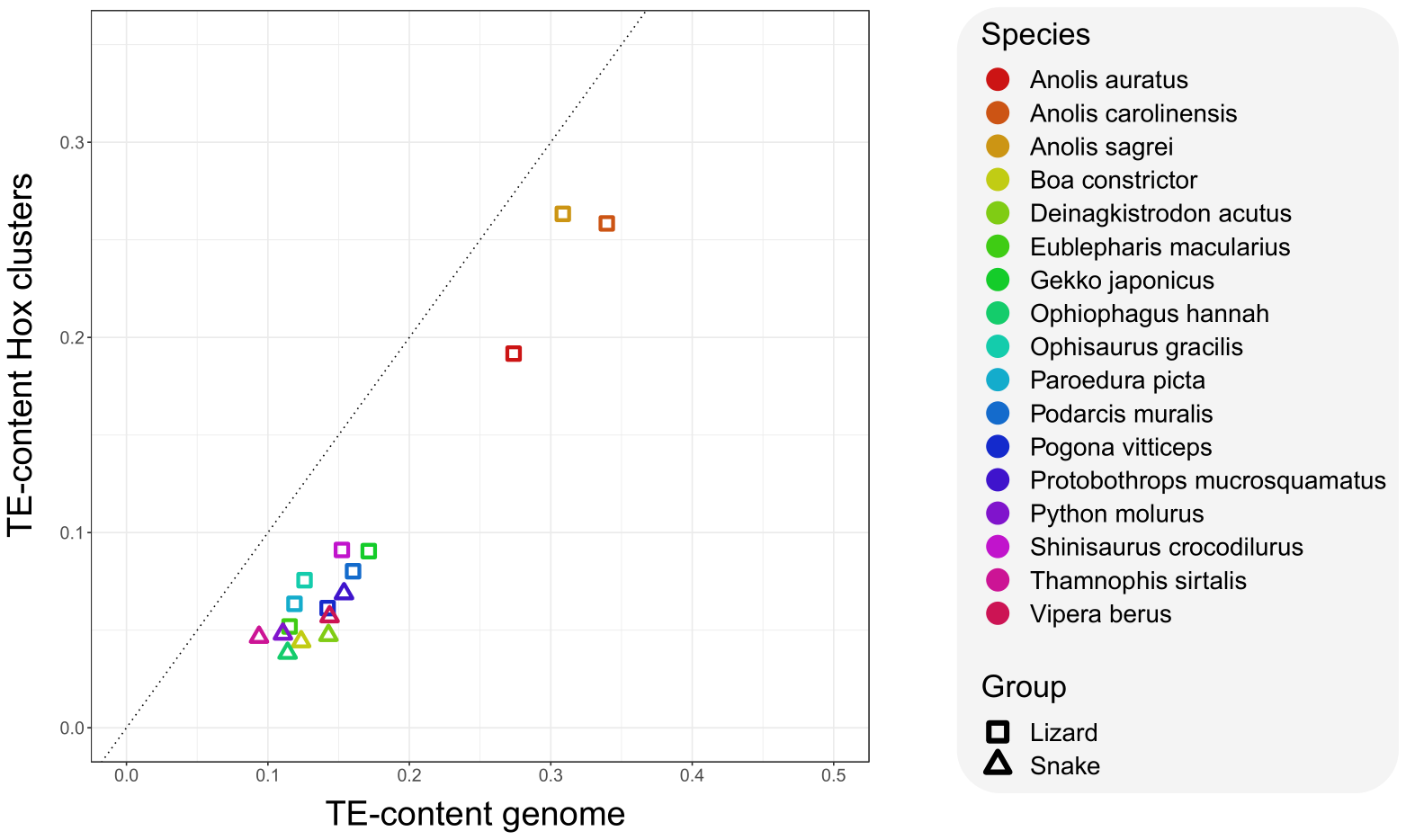

Supplement: Supplementary file 4 — Figure S3. Anolis carolinensis and A. sagrei TE classes across Hox cluster‐containing chromosomal regions. [file EVL3-3-474-s004.tiff]

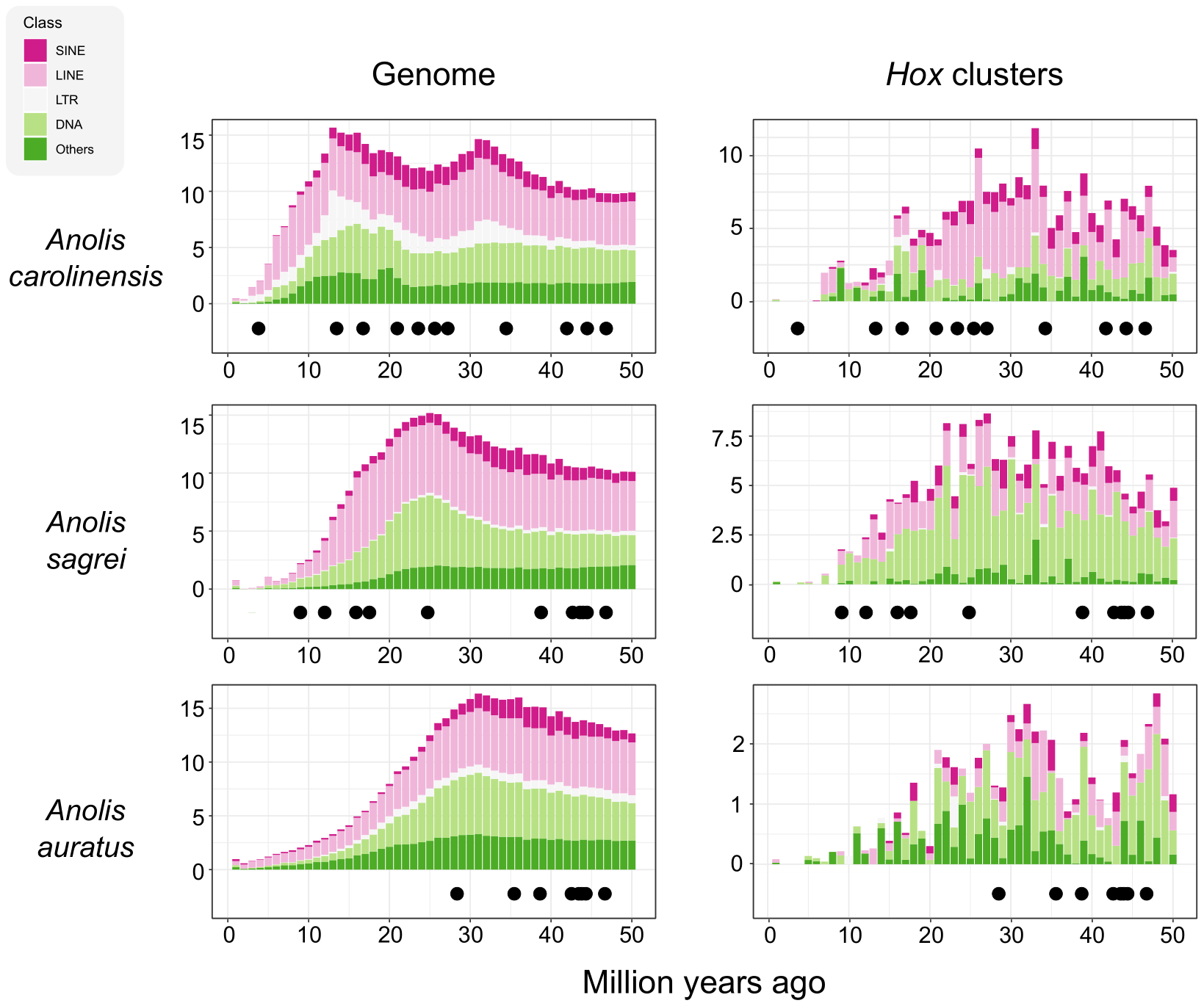

Supplement: Supplementary file 5 — Figure S4. Visualization of pairwise comparison of the four Hox clusters between the wall lizard Podarcis muralis and the green anole Anolis carolinensis. [file EVL3-3-474-s005.tiff]

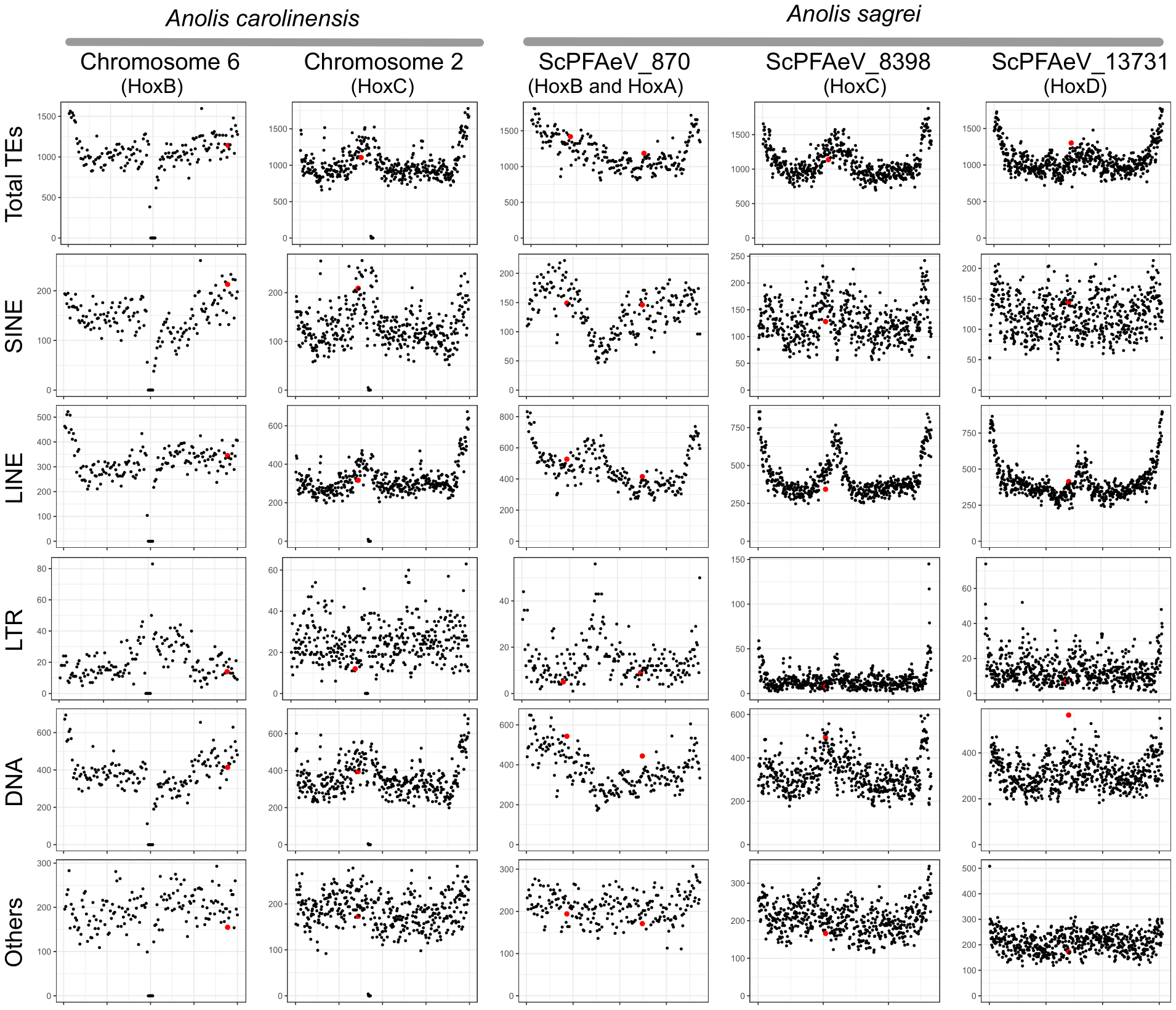

Supplement: Supplementary file 6 — Figure S5. Age of TEs in relation to speciation events in three Anolis species. [file EVL3-3-474-s006.tiff]
